# Functional extracellular matrix hydrogel modified with MSC‐derived small extracellular vesicles for chronic wound healing

**DOI:** 10.1111/cpr.13196

**Published:** 2022-02-14

**Authors:** Shiqing Ma, Han Hu, Jinzhe Wu, Xuewen Li, Xinying Ma, Zhezhe Zhao, Zihao Liu, Chenxuan Wu, Bo Zhao, Yonglan Wang, Wei Jing

**Affiliations:** ^1^ Department of Stomatology The Second Hospital of Tianjin Medical University Hexi District Tianjin China; ^2^ 12610 School and Hospital of Stomatology Tianjin Medical University Tianjin China; ^3^ Beijing Biosis Healing Biological Technology Co., Ltd. Beijing China

## Abstract

**Objectives:**

Diabetic wound healing remains a global challenge in the clinic and in research. However, the current medical dressings are difficult to meet the demands. The primary goal of this study was to fabricate a functional hydrogel wound dressing that can provide an appropriate microenvironment and supplementation with growth factors to promote skin regeneration and functional restoration in diabetic wounds.

**Materials and Methods:**

Small extracellular vesicles (sEVs) were bound to the porcine small intestinal submucosa‐based hydrogel material through peptides (SC‐Ps‐sEVs) to increase the content and achieve a sustained release. NIH3T3 cell was used to evaluate the biocompatibility and the promoting proliferation, migration and adhesion abilities of the SC‐Ps‐sEVs. EA.hy926 cell was used to evaluate the stimulating angiogenesis of SC‐Ps‐sEVs. The diabetic wound model was used to investigate the function/role of SC‐Ps‐sEVs hydrogel in promoting wound healing.

**Results:**

A functional hydrogel wound dressing with good mechanical properties, excellent biocompatibility and superior stimulating angiogenesis capacity was designed and facilely fabricated, which could effectively enable full‐thickness skin wounds healing in diabetic rat model.

**Conclusions:**

This work led to the development of SIS, which shows an unprecedented combination of mechanical, biological and wound healing properties. This functional hydrogel wound dressing may find broad utility in the field of regenerative medicine and may be similarly useful in the treatment of wounds in epithelial tissues, such as the intestine, lung and liver.

## Graphical abstract

Schematic illustration showing synthesis of the SC‐Ps scaffold dressing and nanoscale sEVs loaded SC‐Ps scaffold dressing and the potential application of the dressings in diabetic wound healing and skin reconstruction.

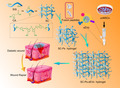

## 1 INTRODUCTION

Impaired wound healing is a global issue that causes a heavy economic and healthcare burden every year, especially with the increasing incidence of diabetes and lengthening of life expectancy.^1^, ^2^ Chronic wounds such as diabetic ulcers, one of the most serious complications of diabetes, involves a constellation of disease processes that are caused or exacerbated by diabetes,^3^ can cause significant disability or even mortality.^4^ Exposure, ischaemia and dehydration often damage the microenvironment of the wound site and interfere with the healing process, particularly in large‐scale and chronic wounds.^5^, ^6^ In addition, due to the absence of a series of specific molecular and cellular processes in the healing process, chronic wounds in diabetic patients take a long time to heal.^7^, ^8^ Therefore, for effective wound healing, an appropriate microenvironment and supplementation with growth factors can ameliorate the local microenvironment in diabetic ulcer patients, promoting skin regeneration and functional restoration.

Currently, although debridement, negative pressure wound therapy (NPWT) and hyperbaric oxygen therapy (HBOT) are used to treat chronic diabetic wounds, their effects are not sufficient.^9^ Compared to gauze, hydrogel dressing serves as a better barrier against trauma and infection, and hydrogel dressings, which are semipermeable to water and oxygen, promote patient function during daily activities and reduce pain from the patient perspective, decrease the frequency of dressing changes, reduce scar tissue formation and promote autolytic debridement.^10^, ^11^ For these reasons, hydrogel dressings are thought to be ideal wound dressings for impaired diabetic wounds. However, conventional hydrogel wound dressings are mostly chemical, and their possible biological toxicity and poor degradability in vivo prevent their widespread use.^12^, ^13^, ^14^ Therefore, finding the source of wound dressing from natural substances has become the focus.

Based on the previous studies of our group on hydrogel wound dressings, we focus on a natural extracellular matrix material, the porcine small intestinal submucosa (SIS). SIS is derived from decellularized porcine jejunum, which is mainly composed of collagen and rich in varieties of biologically active factors.^15^, ^16^, ^17^ SIS can promote the repair and regeneration of self‐tissues in defect areas and has been used as a scaffold material for tissue repair^18^ for its good biocompatibility and low immunogenicity. Moreover, mechanical strength also makes sense for a novel wound dressing, but which is poor of the SIS hydrogel. We have studied that catechol naturally originates from fruits and vegetables and catechol groups can interact with collagen. The introduction of catechol analogues into collagen was shown to improve the properties of collagen.^19^, ^20^, ^21^, ^22^ Therefore, catechol can be introduced to improve the properties of SIS hydrogel.

Umbilical cord mesenchymal stem cells (ucMSCs) possess powerful regenerative and immunomodulatory potential and ucMSC‐derived sEVs have been suggested to be a promising natural source of nanoparticles to improve outcomes in organ transplantation and control inflammatory diseases.^23^, ^24^ Thus, in order to strengthen the ability of promoting wound healing of wound dressing, sEVs can be an excellent supplement to meet the needs of various bioactive molecules in the wound healing process. In the material science field, sEVs are usually directly mixed with scaffold materials.^25^, ^26^, ^27^, ^28^ Simple absorption of sEVs to a scaffold allows the diffusion of sEVs into extracellular fluids and rapid loss of activity, making it difficult to maintain sEVs at a therapeutic concentration in this manner.^29^ High diffusibility and a short half‐life time are weaknesses of direct exogenous delivery and that may greatly reduce the final treatment effect.^30^ Thus, it is necessary to find a new strategy to increase the efficiency of sEVs delivery.

Inspired by fusion peptide technology, which has been applied in the field of functional biomaterial modifications, enables the connection of polypeptide fragments with different functional domains to obtain a new polypeptide with multiple functions. In previous studies, chimeric titanium‐binding peptides with antimicrobial peptides achieved enhanced antimicrobial peptide adsorption on implants compared to non‐specific adsorption.^31^, ^32^

Therefore, we anticipate that the fusion peptides can work as a bridging medium to graft sEVs onto hydrogel materials. In this context, we know that some genetically engineered peptides from collagen and collagen‐binding domains (CBDs, TKKTLRT for collagen I, DARKSEVQK for collagen III) have been screened.^33^ Moreover, the polypeptide fragment CP05 (CRHSQMTVTSRL) identified by phage display was found to specifically recognize CD63, a characteristic protein on the surface of sEVs that can target, load cargo and capture sEVs.^34^ Thus, linking CP05 and CBDs to construct a fusion peptide may be the key to achieving an sEVs targeting self‐assembling hydrogel system.

In this work, a functional hydrogel wound dressing was fabricated based on SIS, sEVs and fusion peptides. Moreover, catecholamine chemistry was used to modify SIS to prepare a hydrogel wound dressing with enhanced biomechanical properties (Graphical Abstract). The experiments demonstrated that the hydrogel wound dressing had good mechanical strength, ability to enhance proliferation, migration and adhesion of fibroblasts and tube formation in vitro. And the functional hydrogel wound dressing was demonstrated through in vivo wound healing experiments by a diabetic full‐thickness skin defect model.

## 2 RESULTS AND DISCUSSIONS

### 2.1 Structure prediction of the fusion peptides

The predicted pseudo‐3D architecture of the four peptides is shown in Figure S1. We constructed two fusion peptides consisting of CBD and CP05 (P1: CBD_I_‐CP05, TKKTLRTCRHSQMTVTSRL; P2: CBD_III_‐CP05, DARKSEVQKCRHSQMTVTSRL) and two scrambled peptides (P3: QTMCTVRTSHRRLTLKSTK; P4: QRQMTCVTVSRLDHAKRKSES) as negative controls. The graphs of mass spectra (MS) and high‐performance liquid chromatography (HPLC) of four peptides are presented in Figures S2 and S3, which show the purity and molecular weight of the peptide. The pseudo‐3D amphipathic structures of the four peptides revealed clusters of secondary structure elements spatially organized into discrete peptide sectors, which illustrated the presence of two helices and a γ‐turn structure in P1 and a β‐turn, γ‐turn and helix structure in P2. According to the hydrophilic/hydrophobic distributions, most of the amino acids in the four peptides are hydrophilic. Thus, all four peptides were considered hydrophilic peptides. Their excellent water solubility allowed the four peptides to form a stable and uniform dispersion system in deionized water and was also the premise for their use in subsequent experiments.

### 2.2 sEVs characterization

**FIGURE 1:**
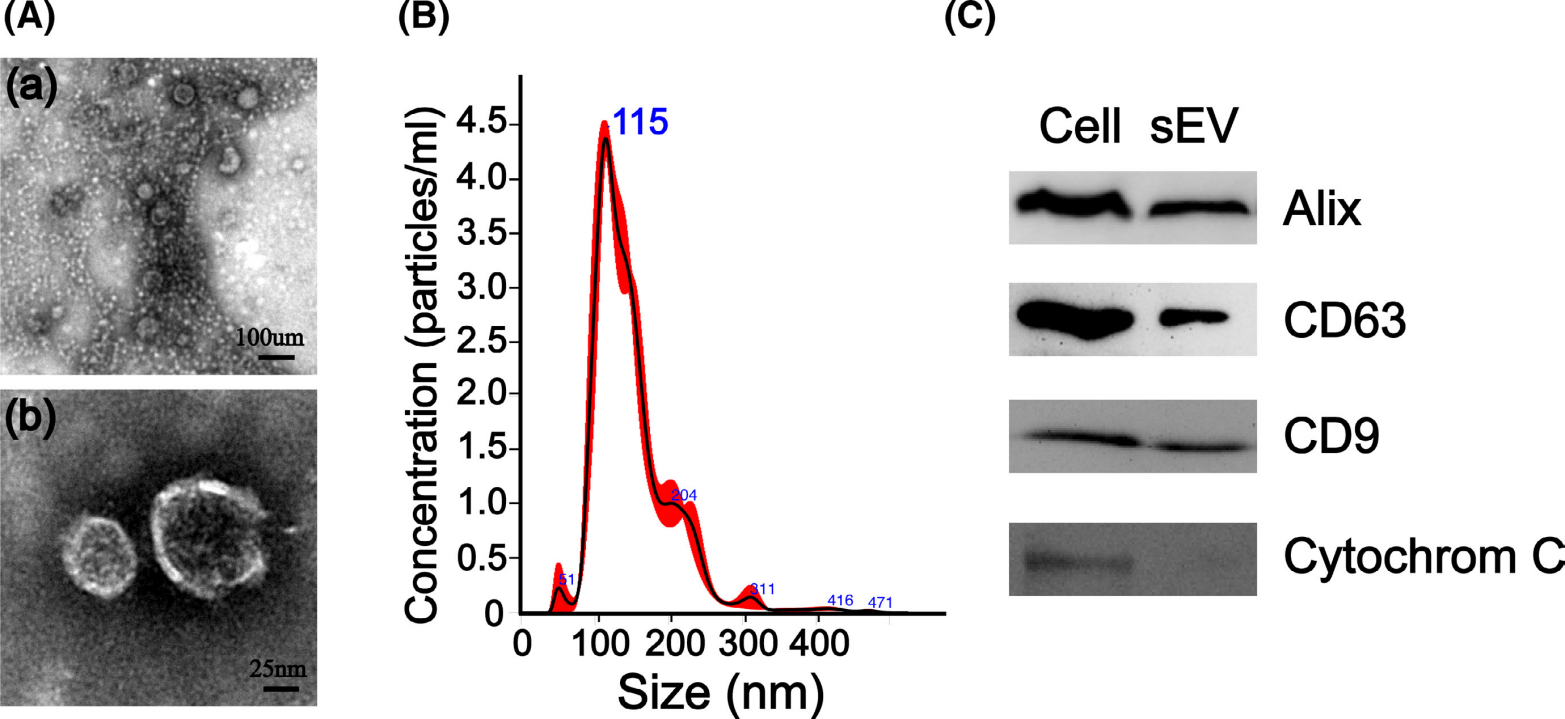
Characterization of ucMSC‐derived sEVs. (A) Morphology of ucMSC‐derived sEVs observed by TEM. (B) Particle size distribution of ucMSC‐derived sEVs analysed by NTA. (C) Western blot analysis of the expression of Alix, CD63, CD9 and cytochrome C in ucMSC‐derived sEVs

sEVs were purified from the culture medium of ucMSCs by ultracentrifugation. Transmission electron microscopy (TEM), nanoparticle tracking analysis (NTA) and western blotting were performed to identify the ucMSC‐derived sEVs. TEM images revealed that the ucMSC‐derived sEVs exhibit a unique shape that resembles a saucer and cup or are round and smaller than 150 nm in size (Figure 1A), consistent with the previously reported morphology of sEVs.^35^ NTA measurements showed that the ucMSC‐derived sEVs are approximately 50–150 nm in size (Figure 1B), which is consistent with the TEM results.^36^ Western blot analysis indicated that the sEVs were positive for the characteristic sEV surface marker proteins Alix, CD9 and CD63 and negative for the specific cytosolic marker cytochrome C (Figure 1C). These results have also been reported in other studies.^23^ Overall, these results indicate that ucMSC‐derived sEVs were successfully obtained in the study. The results of the sEV‐uptake experiment (Figure S4) showed that the intensity and range of red fluorescence increased with time, and no fluorescence was observed in the control group, indicating that the sEVs could be absorbed by NIH3T3 and EA.hy926 cells and that their absorption increased with time.

### 2.3 Physicochemical characterization of the hydrogels

**FIGURE 2:**
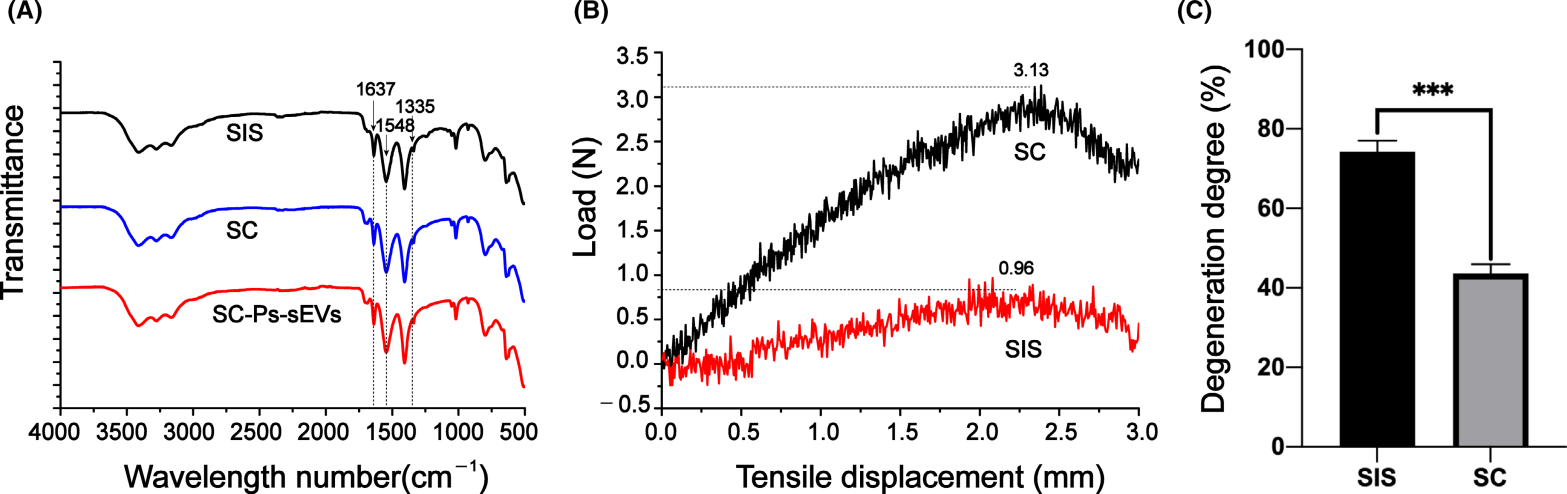
Characterization of hydrogel dressings. (A) FTIR spectra of SIS, SC and SC‐Ps‐sEVs. (B) Tensile stress–strain curves for SIS and SC. (C) Degree of SIS and SC degradation after immersion in a collagenase solution at 37°C for 5 h. The results are the average ± SD, *p < 0.05; **p < 0.01; ***p < 0.005, *n* = 3

A catechol‐modified small intestine submucosa (SC) hydrogel dressing was synthesized using an SIS solution at a concentration of 10% (w/v) and 3‐(3.4‐dihydroxyphenyl) propionic acid by amidation reaction. After the pH had been adjusted to 7.4 and the reaction was incubated at 37°C for 1 h, the SIS and SC hydrogels were obtained. The chemical composition and micromorphology of the SC hydrogel were characterized by Fourier transform infrared spectroscopy (FTIR) and scanning electron microscopy (SEM). Figure 2A depicted the FTIR spectra of SIS and SC. The FTIR spectrum of SIS mainly shows contributions arising from the vibrations of amide groups, and characteristic peaks associated with the major functional groups in collagens were assigned based on a report by Sionkowska.^37^ The SIS spectrum primarily contained amide I (1637 cm^−1^), II (1548 cm^−1^) and III (1335 cm^−1^) bands, which were mainly attributed to C=O vibration, N–H bending and C–N stretching and C–N stretching and N–H in‐plane bending respectively.^22^ The characteristic peak in the SC spectrum did not change significantly after catechol modification, suggesting that the collagen structure was not altered after the introduction of catechol.

**FIGURE 3:**
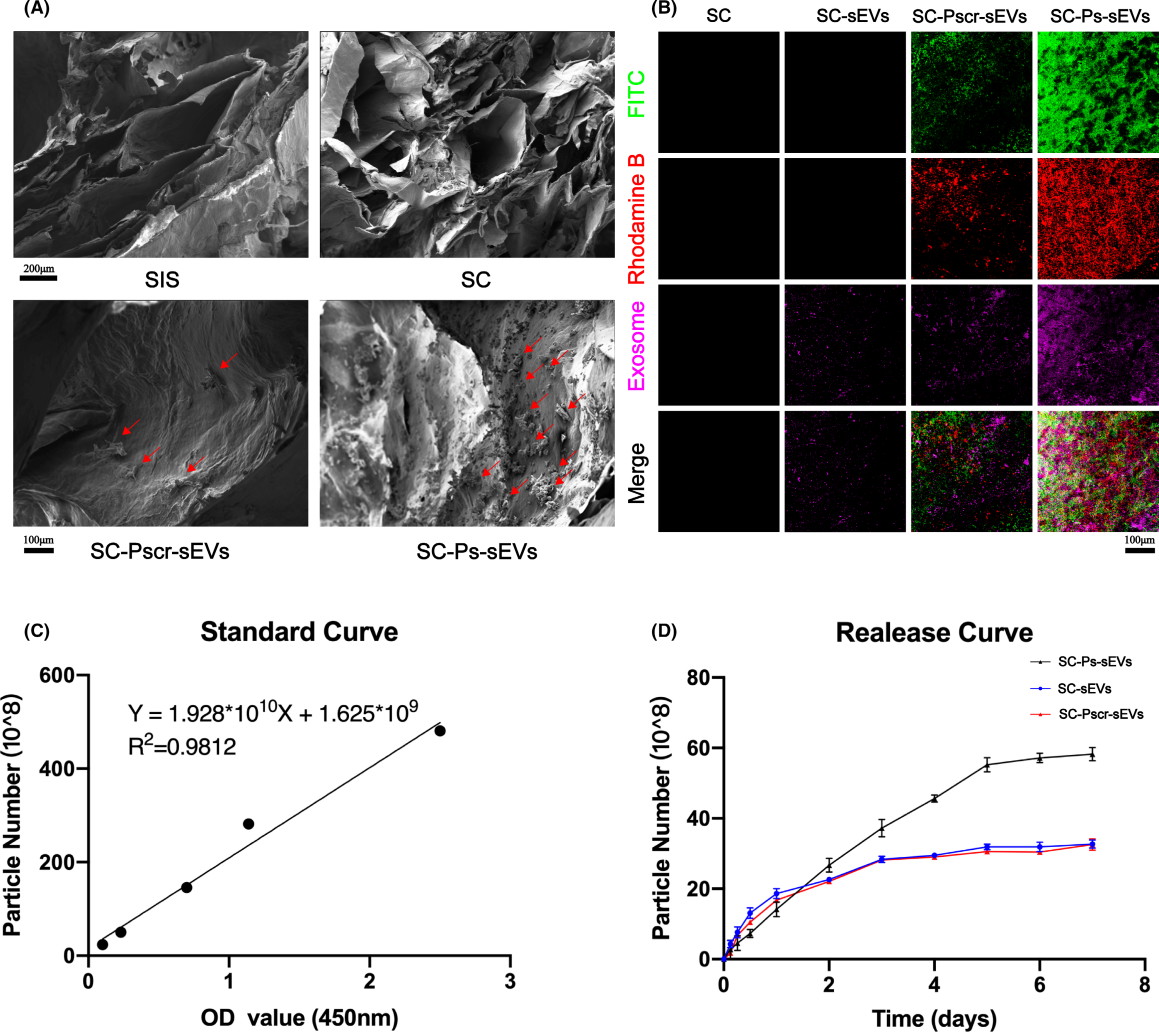
Fusion peptides mediate specific adsorption of the sEVs to SC. (A) SEM images were used to observe the surface morphology in hydrogel samples at different magnifications; the red arrows indicate peptide granules. (B) CLSM images of the SC group, SC‐sEVs group, SC‐Pscr‐sEVs group and SC‐Ps‐sEVs group. SC refers to only SC, SC‐sEVs refers to SC with sEVs, SC‐Pscr‐sEVs refers to SC with two scramble peptides (P3 and P4) and sEVs and SC‐Ps‐sEVs refers to SC with two peptides (P1 and P2) and sEVs. P1 and P3 were labelled with FITC (green), P2 and P4 were labelled with rhodamine B (red) and the sEVs were labelled with DiI (violet). (C) Standard curve from sEV ELISA. (D) ucMSC‐derived sEVs release curves for the SC‐sEVs, SC‐Pscr‐sEVs and SC‐Ps‐sEVs hydrogels. At each time point, five replicates were used. ELISA, enzyme‐linked immunosorbent assay; OD, optical density

In addition, SEM images showed a clearer 3D porous morphology in the SC hydrogel than in the SIS hydrogel (Figure 3A). Moreover, the pores in the SC hydrogel were more compact than those in the SIS hydrogel, suggesting that the SC hydrogel was composed of a denser network of collagen fibres than the SIS hydrogel. Changes in the microstructure lead to changes in macromechanical properties. A mechanical performance test showed that the SC hydrogel had better tensile strength than the SIS hydrogel, and its maximum load was 3.13 N, which was significantly higher than that of the SIS hydrogel (0.96 N) (Figure 2B). After immersion in a collagenase solution at 37°C for 5 h, both hydrogels clearly degraded. As shown in Figure 2C, the degree of enzymatic degradation decreased from approximately 73% for the SIS hydrogel to 42% for the SC hydrogel.

The introduction of catechol did not change the chemical structure of the SIS hydrogel, but significantly increased its mechanical performance and reduced its degradation rate in a collagenase solution. The mechanism in SC hydrogel crosslinking suggests that catechols and catechol quinones were polymerized through a combination of charge‐transfer, π‐stacking, and hydrogen bonding interactions.^38^, ^39^ The unreacted catechols were partly oxidized, and supramolecular aggregates of monomers (consisting of catechols and the oxidized products) were observed. The aggregated supramolecules could form links among the collagen molecules. Additionally, collagen molecules from different aggregates were crosslinked in this manner. Then, a network of collagen aggregates was constructed, and collagen solutions were transformed into hydrogels. FTIR analysis did not show much difference between the SIS and SC hydrogels, as the triple helix structure of collagen was still present after crosslinking. These results are also consistent with the microscopic appearance of the hydrogels. Due to its mechanical enhancement, the SC hydrogel could resist a certain amount of tension during the application process and remained longer on the wound surface than the SIS hydrogel, indicating that the SC hydrogel may play an important role in early closure of the wound.

### 2.4 Characterization of fusion peptide‐mediated specific adsorption

SEM images were used to view the peptides, which are highlighted in the particle images (Figure 3A), the peptides were uniform on the surface of the SC‐Pscr‐sEVs group and SC‐Ps‐sEVs group. The SC‐Ps‐sEVs group contained more particles than the SC‐Pscr‐sEVs group. Meanwhile, introduction of the peptides and exosomes did not change the intrinsic structure of the SC hydrogel (Figure 2A). Confocal laser scanning microscopy (CLSM) showed no fluorescence signals in the SC group (Figure 3B), a small amount of violet fluorescence in the SC‐sEVs group and SC‐Pscr‐sEVs group and almost no difference in intensity between the SC‐sEVs group and SC‐Pscr‐sEVs group. The SC‐Ps‐sEVs group exhibited the most intense violet fluorescence among the groups. In addition, the green and red fluorescence densities of the SC‐Ps‐sEVs group were significantly stronger than those of the SC‐Pscr‐sEVs group. As shown by the SEM results, only a small number of disordered peptides were adsorbed on the surface of the hydrogels, and their presence may have been due to simple physical adsorption. The SC‐Ps‐sEVs group exhibited more peptide particles on the surface of the hydrogel due to the presence of specific collagen‐binding sequences. Similarly, the SC‐Ps‐sEVs group exhibited stronger and denser fluorescence under CLSM, while the SC‐Pscr‐sEVs group also exhibited a small amount of fluorescence due to physical adsorption. Immersion of the SC‐sEVs, SC‐Pscr‐sEVs and SC‐Ps‐sEVs in Dulbecco's modified Eagle's medium (DMEM) for 3, 6 or 12 h or 1, 2, 3, 4, 5, 6 or 7 days followed by dissolution after 7 days of immersion increased the number of sEV particles detected using an sEV ELISA kit. The standard curve is shown in the Figure 3C. The total sEV load of the SC‐Ps‐sEVs hydrogel was 228.07 ± 11.13 × 10^8^ sEV particles, that of the SC‐sEVs was 102.69.65 ± 18.84 × 10^8^ sEV particles and that of the SC‐Pscr‐sEVs was 120.67 ± 16.19 × 10^8^ sEV particles. The release curves are shown in Figure 3D. The total sEVs loads of the SC‐sEVs and SC‐Pscr‐sEVs groups were less than that of the SC‐Ps‐sEVs group, and almost half of the total sEVs had been released on the first day, and all sEVs had been released by the third day. In the SC‐Ps‐sEVs group, the release of sEV particles were slow until the seventh day. Above all, the desired anchoring effect was achieved through use of the fusion peptides, which positioned sEVs on the hydrogel and achieved controlled sEV release. sEVs release was much more controlled with the fusion peptides than release of the sEVs alone through physical adsorption to the hydrogel.

### 2.5 The SC‐Ps‐sEVs hydrogel stimulated the proliferation, migration and adhesion of NIH3T3 cells in vitro

**FIGURE 4:**
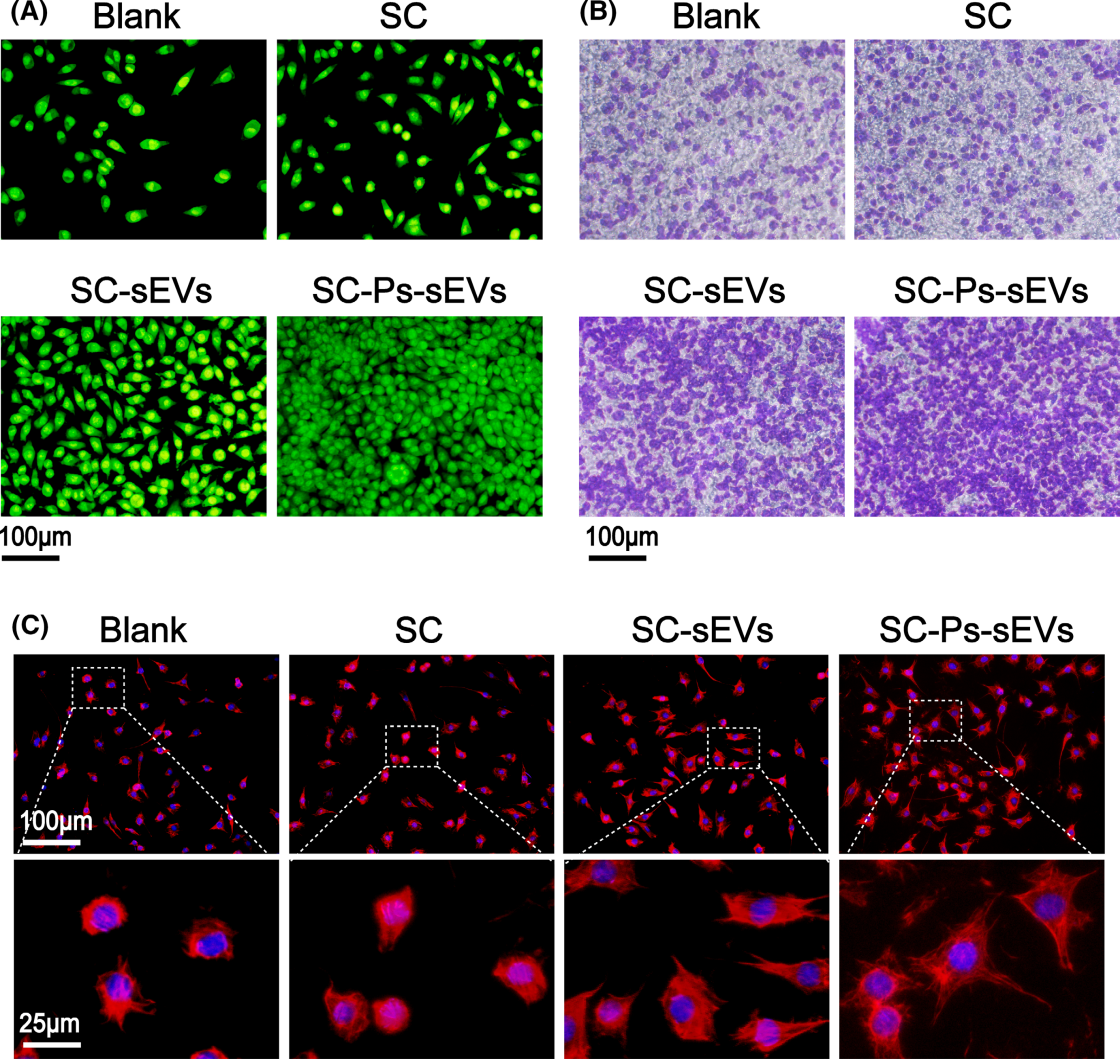
The SC‐Ps‐sEVs hydrogel stimulated the proliferation, migration and adhesion of NIH3T3 cells in vitro. (A) SYTO 9 (green) staining of NIH3T3 seeded on each sample for 3 days. (B) Transwell assay to detect the migration of NIH3T3 cells after 24 h of co‐culture with each sample. (C) Cytoskeleton staining at 36 h after seeding on each sample

**FIGURE 5:**
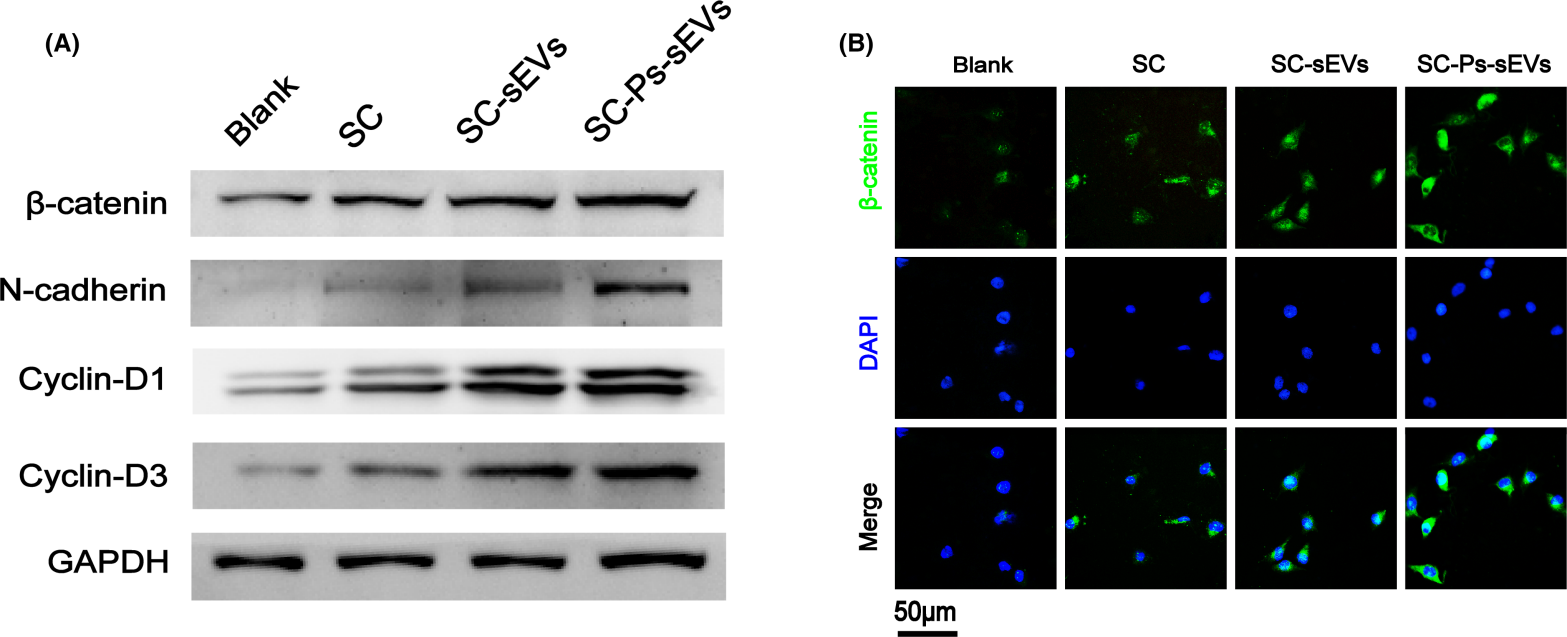
The wnt/β‐catenin pathway was activated by the SC‐Ps‐sEVs hydrogel. (A) Western blot analysis of the expression of β‐catenin and its downstream targets (cyclin‐D1, cyclin‐D3 and N‐cadherin) in NIH3T3 cells treated with PBS and exudates of SC, SC‐sEVs or SC‐Ps‐sEVs at 24 h. (B) The nuclear translocation of β‐catenin (green) in NIH3T3 cells was assessed by IF staining

SYTO 9 staining was used to assess the biocompatibility and proliferation of fibroblasts after seeding on blank wells, SC, SC‐sEVs and the SC‐Ps‐sEVs hydrogels for 3 days (Figure 4A and Figure S5A). Cells in each group proliferated over time. Based on the results, the most live cells were observed in the SC‐Ps‐sEVs group. Compared to that in the blank well, the SC group showed no obvious difference in cell number. The cell number in the SC‐sEVs group was obviously greater than that in the SC and control groups, but not as high as that in the SC‐Ps‐sEVs group. This finding indicates that the SC‐Ps‐sEVs hydrogel strongly promoted cell survival and proliferation. Transwell assays were carried out to assess the migration of fibroblasts incubated with different samples for 24 h (Figure 4B and Figure S5B). In the SC‐Ps‐sEVs group, the number of cells that passed through the membrane was remarkably higher than that of the blank well, SC group and SC‐sEVs group. The morphology and distribution of fibroblasts seeded on blank wells, SC, SC‐sEVs and SC‐Ps‐sEVs hydrogels for 36 h were evaluated by cytoskeleton staining and observed under CLSM (Figure 4C). Confocal images showing that fibroblasts adhered on the SC‐Ps‐sEVs and SC‐sEVs hydrogels exhibit a stellate phenotype, whereas those on the blank well and SC hydrogel were oval, a morphological feature indicating non‐adherence. In addition, cells on the SC‐sEVs and SC‐Ps‐sEVs hydrogels exhibited well‐stretched actin bundles (red) with clear lines consisting of actin filaments. Additionally, the cells, especially those in the SC‐Ps‐sEVs group, extended more pseudopods than those in the other groups. A previous study reported that ucMSC‐derived sEVs can enhance cell proliferation and migration by activating the wnt/β‐catenin pathway to promote wound healing.^23^, ^40^ Western blot analysis (Figure 5A and Figure S6) was used to determine the expression of proteins in the wnt/β‐catenin signalling pathway, including β‐catenin and β‐catenin downstream targets N‐cadherin, cyclin‐D1 and cyclin‐D3. The expression of β‐catenin, N‐cadherin, cyclin‐D1 and cyclin‐D3 was significantly increased in the SC, SC‐sEVs and SC‐Ps‐sEVs groups compared with the control group and was highest in the SC‐Ps‐sEVs group. These results suggest that the ucMSC‐derived sEVs bound to the SC hydrogel through the fusion peptides could trigger the wnt/β‐catenin signalling pathway and up‐regulate the expression of downstream proteins. To directly observe the effects of the SC‐Ps‐sEVs hydrogel on the wnt/β‐catenin pathway, we examined β‐catenin levels using immunofluorescence (IF). As shown in Figure 5B, β‐catenin (green) was distributed in the cytoplasm and nuclei of NIH3T3 cells after 24 h of incubation. β‐Catenin levels in the cytoplasm and nucleus were obviously greater in the SC‐Ps‐sEVs group than in the other three groups. Only a small amount of β‐catenin was observed in the cytoplasm, and hardly any was found in the nucleus in the control group. The SC group showed more β‐catenin expression than the control group, but less β‐catenin expression than the SC‐sEVs and SC‐Ps‐sEVs groups. This may be because the binding of wnt family signalling molecules carried in sEVs to target cell receptors activated the wnt/β‐catenin pathway, causing β‐catenin molecules to accumulate in the cytoplasm and enter the nucleus.

### 2.6 Evaluation of tube formation in vitro

**FIGURE 6:**
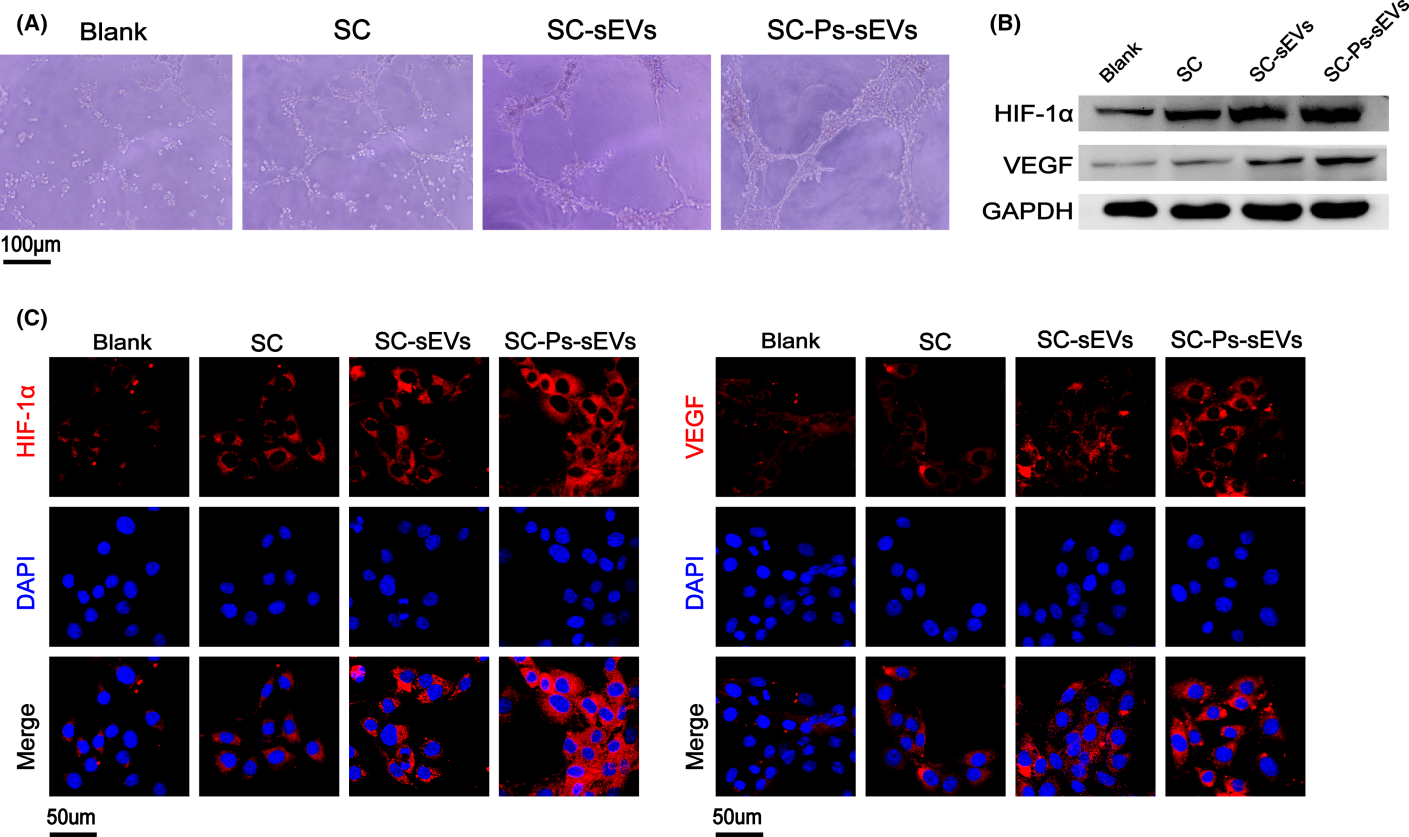
Angiogenesis was promoted in vitro. (A) Tube formation in EA.hy926 cells treated with PBS and exudates of SC, SC‐sEVs and SC‐Ps‐sEVs on Matrigel for 8 h. (B) Western blot analysis to assess the HIF‐1α and VEGF content in EA.hy926 cells treated with PBS and exudates of SC, SC‐sEVs or SC‐Ps‐sEVs for 24 h. (C) IF staining of HIF‐1α (red) and VEGF (red) in EA.hy926 cells treated with PBS and exudates of SC, SC‐sEVs or SC‐Ps‐sEVs for 24 h

Tube formation assays using EA.hy926 cells were carried out to evaluate the proangiogenic potential of the SC‐Ps‐sEVs hydrogel. After incubation on Matrigel substrate for 8 h, EA.hy926 cells incubated with control medium, SC and SC‐sEVs formed sparse or even incomplete tube networks, and enhanced tube formation was observed in the SC‐Ps‐sEVs group, which was characterized by an increased tube number compared to those in the control, SC and SC‐sEVs groups and a complete tubular structure (Figure 6A and Figure S7A).

To evaluate the ability of the SC‐Ps‐sEVs hydrogel to promote angiogenesis at the protein level, western blot analysis and IF staining were used. The western blotting results showed that expression of the angiogenic marker proteins HIF‐1α and VEGF was significantly increased in the SC, SC‐sEVs and SC‐Ps‐sEVs groups compared with the control group, and the highest expression was in the SC‐Ps‐sEVs group (Figure 6B and Figure S7B). As shown in Figure 6C, HIF‐1α (red) and VEGF (red) were mainly distributed in the cytoplasm. The HIF‐1α and VEGF levels were greater in the SC‐Ps‐sEVs group than in the control, SC and SC‐sEVs groups. The IF staining results revealed similar HIF‐1α and VEGF levels in the four groups, as shown by western blot analysis. These findings indicated that the SC‐Ps‐sEVs hydrogel could activate the HIF‐1α/VEGF pathway and promote angiogenesis in vitro, exerting a potent effect on angiogenesis This may play an active role in the healing of chronic wounds.^41^ Mesenchymal stem cells (MSCs) have potent proangiogenic properties that have been attributed to their secretion of paracrine factors. There may be multiple mechanisms involved in the modulation of angiogenesis by MSC‐derived EVs. In addition to direct delivery of VEGF,^42^ the platelet‐derived growth factor‐D (PDGF‐D) had been identified to be an important player in the EV‐mediated stimulation of angiogenesis.^43^ The activation of the wnt4/β‐catenin pathway was also reported as another mechanism involved in the proangiogenic effect of ucMSC‐derived EVs.^44^

### 2.7 Assessment of diabetic wound healing in vivo

**FIGURE 7:**
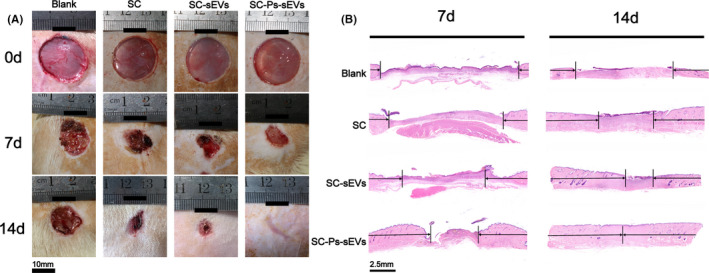
Healing process and histological evaluation of diabetic wound healing by the SC‐Ps‐sEVs hydrogel dressing. (A) Representative images of full‐thickness skin defects at 0, 7 and 14 days after surgery treated with control, SC, SC‐sEVs and the SC‐Ps‐sEVs hydrogel dressing. (B) Representative images of H&E‐stained wound samples on days 7 and 14 from groups treated with SC, SC‐sEVs and SC‐Ps‐sEVs hydrogel dressing and the control group

The above results proved that the SC‐Ps‐sEVs hydrogel possesses favourable properties and good biocompatibility and thus should be suitable for diabetic wounds. Therefore, we examined the wound healing ability of the SC‐Ps‐sEVs hydrogel dressing using a full‐thickness diabetic wound model. Figure 7A shows images obtained by gross observation of the wounds at different time points. None of the wounds showed external evidence of infection during the healing period. The wound areas in all groups were clearly reduced after 7 days of treatment, and the wounds in the SC‐Ps‐sEVs group showed the fastest healing among the groups. Compared with wound in the SC and SC‐sEVs groups, wounds in the SC‐Ps‐sEVs group had healed to the greatest extent 2 weeks later and were nearly completely covered with newly formed skin; however, wounds in the SC and control groups were still exposed and covered by eschar, and wounds in the SC‐sEVs group showed better healing compared to those in the SC and control groups. The wound closure rate also confirmed the results of gross observation (Figure S8A). At the early healing period on day 7, the SC‐Ps‐sEVs group showed the highest wound closure rate, followed by the SC‐sEVs group. These results indicated that the sEVs loaded in the fusion peptide‐modified SC scaffold dressing not only maintained their function in vitro, but also play an important role in the repair and regeneration of chronic cutaneous wounds.

We further performed haematoxylin–eosin (H&E) and Masson's trichrome staining of wound samples to evaluate their histological status during the healing period on days 7 and 14. Thick, abundant granulation tissue was clearly seen in the SC‐Ps‐sEVs hydrogel‐treated wounds (Figure 7B), however, wounds in the control group showed a very small amount of newly formed tissue on day 7. Wounds in the SC‐sEVs group also showed good healing with granulation tissue, and more granulation tissue was observed in this group than in the SC group. Statistical analysis of the wound length also confirmed the observation from the H&E‐stained images (Figure S8B); the shortest wound length was observed in the SC‐Ps‐sEVs group, followed by the SC‐sEVs, SC and control groups. After 2 weeks of treatment, the wounds in all groups had healed considerably and were covered with neoepidermis. However, the thickness and differentiation of the neoepidermis and length of the wound area differed among the groups. A significantly decreased wound length was observed in the SC‐Ps‐sEVs group, along with skin appendages in the scar tissue. Other wounds in the SC, SC‐sEVs and control groups showed a much longer wound area without skin appendages. These H&E staining results suggested that the wounds treated with the SC‐Ps‐sEVs hydrogel achieve relatively satisfactory healing with not only a fast healing rate, but also the presence of skin appendages in the scar area.

**FIGURE 8:**
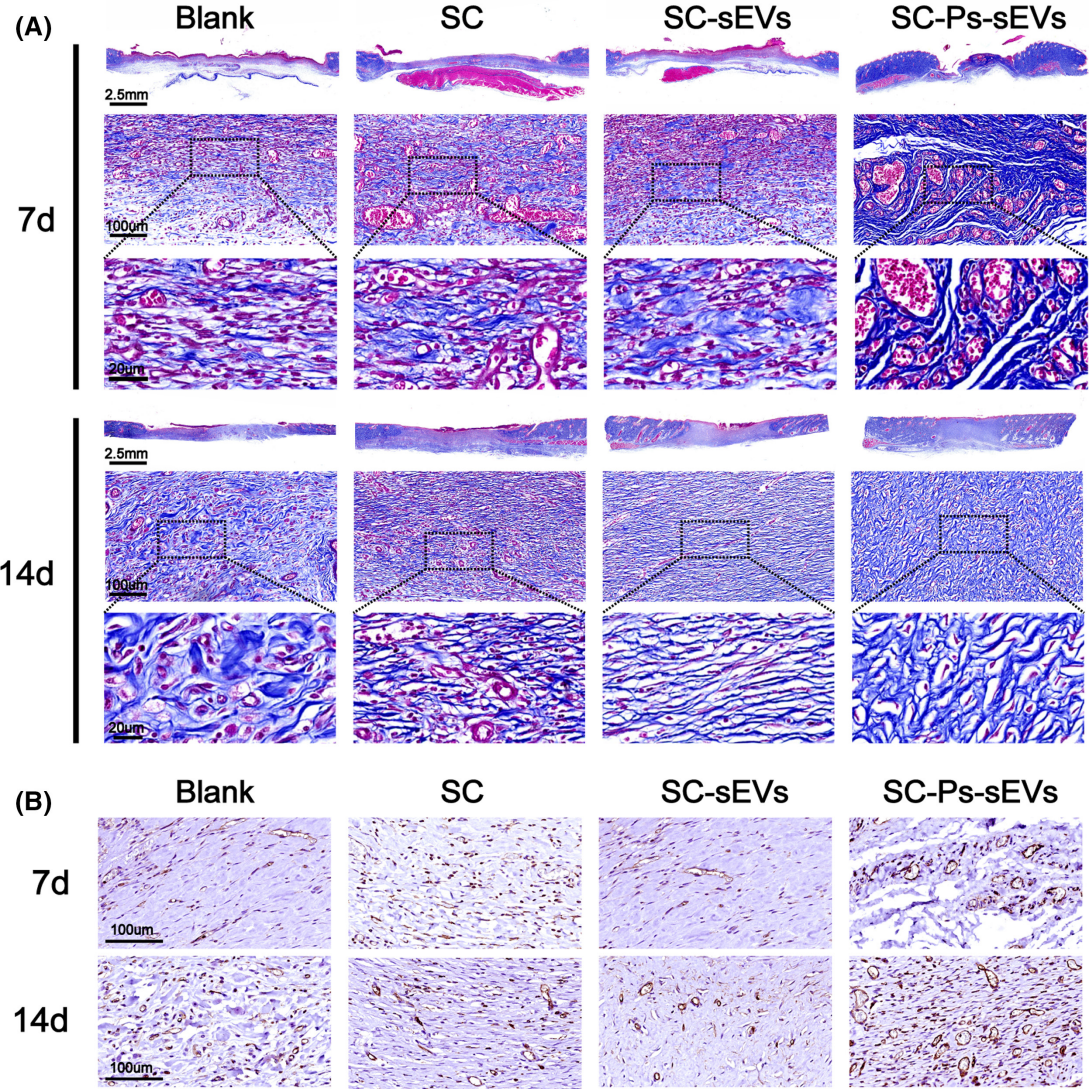
Evaluation of collagen deposition and neovascularization. (A) Images of Masson's trichrome‐stained sections on days 7 and 14 after surgery. (B) Immunostaining of CD34 on days 7 and 14 after surgery

In addition, Masson staining revealed collagen deposition in the wounds treated with the different agents (Figure 8A). On day 7 after operation, few collagen fibres were seen in the control and SC hydrogel‐treated wounds. The SC‐sEVs group showed more regenerated collagen than these two groups, and the most collagen was found in the SC‐Ps‐sEVs group. With prolonged healing time, on day 14, the amount of newly formed collagen in all groups had increased. Despite this increase, the collagen fibres in the SC‐Ps‐sEVs and SC‐sEVs groups were more organized than those in the SC and control groups, but a denser scar structure was observed in the SC‐sEVs group than in the SC group. Moreover, skin appendages could clearly be seen in the SC‐Ps‐sEVs group, confirming the above results of H&E staining. Together, these histological results provided by H&E and Masson staining indicated that the SC‐Ps‐sEVs hydrogel dressing is very suitable for diabetic wounds and promotes granulation tissue formation and collagen formation, generates skin appendages in the wound and ultimately accelerates the healing process in diabetic wounds.

Blood vessels are thought to be critical for tissue regeneration due to their function in providing nutrition and oxygen to cells around wounds. From the in vitro results, we found that the SC‐Ps‐sEVs hydrogel strongly promoted angiogenesis in EA.hy926 cells (Figure 6). Nevertheless, whether the ability of the SC‐Ps‐sEVs hydrogel to promote angiogenesis in vitro would affect angiogenesis in diabetic wounds remained unclear. Therefore, the level of CD34 was assessed to evaluate newly formed vessels within the regenerated tissue. Immunostaining of CD34 in wound samples was performed on days 7 and 14 to investigate whether angiogenesis could be activated in diabetic wounds in vivo. As shown in Figure 8B, very little positive staining was found in the control group. The SC‐Ps‐sEVs group showed the strongest positive CD34 signal, while the SC‐sEVs and SC groups showed a small amount of positive staining. Newly formed vessels were also counted, and the results are listed in Figure 8B. These results indicated that the SC‐Ps‐sEVs hydrogel dressing could efficiently activate the angiogenesis process in vitro and in vivo in diabetic wounds.

Polymer materials can undergo structural destruction, and their performance can change in biological environments, and their degradation products can be excreted or absorbed through normal metabolism. In a salt solution, SC was found to absorb water, expand and initially precipitate.^45^ In this swelling process, the material becomes larger in volume, voids in the structure are produced and liquid enters the SC, creates conditions conducive to the further hydrolysis of SC. Upon the degradation of collagen scaffolds, sEVs adsorbed on SC are slowly released and diffuse into bodily fluids or dissolution media to exert their significant effect in promoting wound healing at the wound site until SC is completely degraded. In the process of material degradation, sEVs originally anchored on collagen are released into the surrounding microenvironment and taken up by target cells to promote wound healing.

Paracrine sEVs are thought to be the main mediator of the effects of MSCs on tissue repair. Early evidence showed that sEVs derived from ucMSCs containing abundant Wnt4 protein could activate the β‐catenin signalling pathway to promote the rebuilding of new vessels and collagen synthesis after injury, and these types of extracellular vesicles were also shown to impact the migration of keratinocytes, contributing to acceleration of the re‐epithelialization process via activation of the AKT/GSK3b pathway. Moreover, the abundant mRNAs identified by high‐throughput transcriptome sequencing, such as miR‐23a and miR‐21, can inhibit collagen deposition by blocking the TGF‐β/SMAD2 signalling pathway, and ucMSC‐derived sEVs can reduce the excessive accumulation of myofibroblasts after skin injury and inhibit scar formation. ucMSC‐derived sEVs can also deliver many factors, such as G‐CSF, PDGF‐BB, VEGF, MCP‐1, IL‐6 and IL‐8, to accelerate healing.^23^, ^46^, ^47^

In conclusion, a fusion peptide‐modified SC hydrogel into which sEVs were incorporated shows great potential in promoting diabetic wound healing, and strong evidence for the future clinical application of ucMSC‐derived sEVs is also provided. sEVs can be easily internalized by target cells through the caveolae pathway due to their excellent properties, including their cup‐like or round shape and lipid bilayer membrane, nanometre size and suitable density, which protect them from endosome‐ or lysosome‐induced degradation and maintain their functional components. Therefore, sEVs show great promise in nanomedicine and other opportunities. Clinical research on sEV therapy has been carried out for many years. However, related drugs have not yet been approved, which may be due to an insufficient understanding of sEVs and their components. However, standard purification, storage and administration procedures for the low‐cost clinical application of ucMSC‐derived sEVs in wound healing should be developed. In addition, further examination of whether the therapeutic effects of sEVs are determined by their total composition or by specific components is needed to exclude possible side effects. Multifunctional bioactive biomaterials that carry out long‐term sEV release is also very important for synergistically enhancing tissue regeneration and treatment.

## 3 CONCLUSION

In summary, a functional hydrogel wound dressing with good mechanical properties, excellent biocompatibility and superior stimulating angiogenesis capacity was designed and facilely fabricated, which could effectively enable full‐thickness skin wounds healing in diabetic rat model. This work led to the development of SIS, which shows an unprecedented combination of mechanical, biological and wound healing properties. This functional hydrogel wound dressing may find broad utility in the field of regenerative medicine and may be similarly useful in the treatment of wounds in epithelial tissues, such as the intestine, lung and liver.

## 4 EXPERIMENTAL SECTION

### 4.1 Synthesis of fusion peptides

Four peptides were commercially synthesized (Jill Biochemistry Co., Ltd., Shanghai, China) and are listed in Table 1.^48^ The fusion peptides used for CLSM were labelled with FITC or rhodamine B. The peptides were purified to at least 95% purity and analysed using mass spectrometry (MS) and high‐performance liquid chromatography (HPLC). Peptide solutions were prepared by dissolving the peptides in double distilled water. The 3D molecular architecture of the four peptides was analysed with the protein analysis software VMD. I‐TASSER (https://zhanglab.ccmb.med.umich.edu/I‐TASSER/) was used to predict the secondary structure of the peptides.

**TABLE 1:** Sequences of the fusion peptides

Peptide no.	Composition	Sequence
P1 (FITC)	CBD_I_ + CP05	TKKTLRTCRHSQMTVTSRL
P2 (rhodamine B)	CBD_III_ + CP05	DARKSEVQKCRHSQMTVTSRL
P3scr (FITC)	(CBD_I_ + CP05) scr	QTMCTVRTSHRRLTLKSTK
P4scr (rhodamine B)	(CBD_III_ + CP05) scr	QRQMTCVTVSRLDHAKRKSES

### 4.2 Isolation and characterization of sEVs

ucMSCs (Qincheng Biotechnology Co., Ltd., Shanghai, China) were cultured in DMEM (Gibco) supplemented with 10% sEV‐free foetal bovine serum (FBS, Gibco) and 1% penicillin and streptomycin (Gibco) at 37°C in an atmosphere of 5% CO_2_, after which the cultures were centrifuged at 10,000×*g* for 8 h to isolate the sEVs. The culture medium was collected when the cells reached 80–85% confluency and preserved at −80°C.

sEVs were extracted and purified by differential ultracentrifugation as previously described.^49^ Briefly, the cell culture medium was centrifuged at 500×*g* for 20 min, followed by centrifugation at 3000×*g* for 20 min and 20,000×*g* for 1 h at 4°C. Then, the supernatant was passed through a 0.22‐μm filter (Millex) to remove cell debris. sEVs were obtained by ultracentrifugation at 100,000×*g* for 4 h. The sEVs were resuspended in PBS and recovered by centrifugation at 100,000×*g* for 1 h. The sEVs were then dissolved in 50 μl of PBS. The sEVs were then stored at −80°C. The protein content of the sEVs was determined with a BCA protein assay kit (Thermo Fisher Scientific) and used to quantify the sEVs.

The morphology of the extracted sEVs was observed using TEM (Hitachi, HT7700). Briefly, 10‐μl volumes of the sEVs were pipetted onto a grid coated with formvar and carbon at room temperature and incubated for 10 min, after which the excess fluid was removed. Then, the samples were negatively stained with 3% phosphoric acid (pH 6.8) for 5 min. The size distribution of the sEVs was analysed by NTA (Malvern, NS300). Alix, CD63 and CD9, which are frequently located on the surface of sEVs, were analysed using western blot analysis, and cytochrome C, which is not expressed on sEVs, was analysed using western blot analysis as a negative control.

To examine the internalization of ucMSC‐derived sEVs by NIH3T3 and EA.hy926 cells, the sEVs were labelled with DiI (Beyotime, China). Unlabelled sEVs were used as the control group. Then, the labelled and unlabelled sEVs were incubated with NIH3T3 and EA.hy926 cells for 2, 6 and 12 h. After that, the cells were fixed in 4% paraformaldehyde and washed twice with PBS. The nuclei were stained with DAPI (Thermo Fisher Scientific) and then observed by CLSM.

### 4.3 Preparation of hydrogel materials

Porcine SIS was prepared using a previously reported method.^50^ Briefly, the porcine jejunum was harvested from market pigs within 4 h of sacrifice. After fat was removed through mechanical removal of the tunica, serosa and tunica muscularis, SIS was extracted from the porcine jejunum and then carefully washed with a saline solution. After washing, the SIS was freeze‐dried at −80°C for 48 h using a freeze dryer. The dried SIS was pulverized using a freezer mill at −198°C to yield SIS powder with particles 10–20 µm in size. The obtained SIS powder was stirred for 48 h in an aqueous solution consisting of 3% acetic acid and 0.1% pepsin (25°C), and the final SIS digestion solution was freeze‐dried again at −80°C to obtain a soluble SIS matrix. The obtained SIS matrix was sterilized using ethylene oxide gas.

Then, to form a gel, the soluble SIS matrix was dissolved in phosphate‐buffered saline (PBS) and neutralized to a pH of 7.4 with the addition of 2.5 M NaOH. Then, the neutralized solution was placed in an incubator heated to 37°C for 1 h, and a gel formed. SIS gel at concentrations of 10 mg/ml was prepared. To prepare catechol‐modified SIS gel (SC), before neutralization, 50 mM 3‐(3.4‐dihydroxyphenyl) propionic acid was introduced and stirred for 1 h at room temperature. After crosslinking, the scaffolds were rinsed with PBS twice for 24 h and rinsed with DMEM four times for 15–20 min.

The SC hydrogel was incubated overnight with a 0.05 mg/ml fusion peptide solution and sEVs at 4°C and then rinsed three times with PBS for 10 min. The SC hydrogel directly loaded with sEVs was labelled SC‐sEVs, the SC hydrogel combined with P1, P2 and sEVs was labelled SC‐Ps‐sEVs and the SC hydrogel combined with P3, P4 and sEVs was labelled SC‐Pscr‐sEVs. The hydrogels were prepared in 6‐well plates and 24‐well plates (Corning, NY, USA) for cell assays in vitro and in vivo.

### 4.4 Characterizations of the hydrogel materials

The samples were freeze‐dried and then analysed by FTIR and SEM. For FTIR, the functional groups of the samples were detected with an FTIR spectrophotometer (Bruker Daltonics, Billerica, MA) using the KBr pellet technique. For SEM, freeze‐dried samples were sprayed with gold. Morphological images of the samples were acquired on a scanning electron microscope (Carl Zeiss, Germany). For the tensile strength test, the mechanical properties of the SIS and SC hydrogels were assessed using a universal mechanical machine (INSTRON 1121, USA). For the degradation assay, collagenase (Sigma) was added to Tris/HCl buffer (50 mmol/L) containing 0.36 mmol/L CaCl_2_ to 30 U/ml. The samples were incubated in 50 ml of distilled water (replaced twice a day) at room temperature for 3 days to remove residual reagents. Then, the hydrogels were accurately weighed (W0) and immersed in 15 ml of an enzyme buffer solution at 37°C. After 5 h, the degradation was terminated, and the hydrogels were accurately weighed (W1). The degree of degradation was calculated with the following equation: degree of degradation (%) = (1 − W1/W0) × 100. The degree of degradation was calculated from three replicates. The sEV particle number was detected with a CD63 ExoELISA kit (System Biosciences, USA). Briefly, a quantitative standard curve and a cumulative controlled release curve were determined with a microplate reader (BioTek, USA). The hydrogels loaded with ucMSC‐derived sEVs were immersed in DMEM for 3–12 h and 1–7 days. After 7 days of immersion, the residual sEVs in the hydrogels were also dissolved, and the total number of sEVs was calculated.

### 4.5 Assay to detect fusion peptide‐mediated specific binding of sEVs to SC

To detect the specific binding of sEVs to SC, samples were observed by CLSM (Carl Zeiss, Germany), and images were obtained using an Axiocam digital camera (Carl Zeiss, Germany). As shown in Table 1, P1 and P3 were labelled with FITC, and P2 and P4 were labelled with rhodamine B. sEVs were labelled with DiI (Beyotime, China).

### 4.6 Cell viability and migration assays and cytoskeleton staining

Fibroblast cells are directly involved in the skin tissue regeneration and re‐capitalization process, the NIH3T3 cell line has been used as an in vitro model cell for studying wound healing.^51^, ^52^, ^53^, ^54^ NIH3T3 cells at passages 4–6 were used in our study and cultured on hydrogels in DMEM supplemented with 1% penicillin–streptomycin and 10% FBS. The viability of the cells was measured with a SYTO 9 staining kit (Beyotime, China). Briefly, NIH3T3 cells were seeded on SC, SC‐sEVs and SC‐Ps‐sEVs. The cells were cultured for 3 days and stained with a SYTO 9 staining kit. Then, they were visualized by CLSM. Three replicates were examined for each group. Transwell assays were carried out to assess migration of the NIH3T3 cells. Briefly, 1 × 10^4^ cells/well were cultured in medium containing 5% FBS and seeded into the upper chamber of 24‐well Transwell plates (Corning). Then, complete medium containing 10% FBS supplemented with SC, SC‐sEVs and the SC‐Ps‐sEVs hydrogels was added to the lower chamber. After 24 h, the Transwell chambers were removed and washed twice with PBS. Then, the cells were fixed with methanol for 30 min and stained with 0.1% crystal violet (Sigma‐Aldrich) for 20 min. Cells that did not migrate were gently wiped off with a cotton swab, and the plate was washed with PBS 3 times. Migration was observed under an optical microscope (Leica DM16000B, Germany). To observe the cytoskeleton of cells in the different groups, the hydrogels were seeded with NIH3T3 cells in 24‐well plates (1 × 10^4^ cells/well). After 36 h, the cells were fixed in a 4% formalin solution for 30 min and incubated with 0.5% Triton X‐100 (Beyotime, China) for 10 min. Actin staining was performed to visualize the cytoskeleton through rhodamine B phalloidin (Cytoskeleton, Inc.). DAPI (Thermo Fisher Scientific) was used to stain the cell nuclei. Cellular morphology was observed by CLSM.

### 4.7 In vitro tube formation assay

In vitro capillary network formation in Matrigel matrix (BD Biosciences, San Jose, CA) was monitored by evaluating the effects of the hydrogels on the tube‐formation activity of EA.hy926 cells.^55^, ^56^ The cells were seeded on Matrigel in 96‐well plates at a density of 8000 cells/well, and PBS and solutions extracted from the SC, SC‐sEVs and SC‐Ps‐sEVs were added to each group. Tube formation was observed under an optical microscope.

### 4.8 Western blotting

Solutions extracted from the SC, SC‐sEVs and SC‐Ps‐sEVs were used to treat cells seeded on dishes. When the cells reached the expected density, they were trypsinized (0.05% trypsin/ethylenediaminetetraacetic acid solution, Gibco) and washed twice with PBS. The primary antibodies used were anti‐β‐catenin (1:1000, Abcam), anti‐N‐cadherin (1:1000, Abcam), anti‐cyclin‐D1 (1:1000, Abcam), anti‐cyclin‐D3 (1:1000, Abcam), anti‐VEGF (1:1000, Abcam), anti‐HIF‐1α (1:1000, Abcam) and anti‐GAPDH (1:1000, Abcam) (Abcam, Cambridge, UK). Densitometric quantification of the band intensities was carried out with Image‐Pro Plus 6.0 software, and the relative expression level of each target protein was normalized to the intensity of the GAPDH band.

### 4.9 IF staining

Solutions extracted from the SC, SC‐sEVs and SC‐Ps‐sEVs were used to treat cells seeded on dishes. The cells were fixed in 4% formaldehyde for 10 min before being permeabilized in 0.5% (v/v) Triton X‐100 for 3 min. The samples were washed another two times with PBS and then blocked in a 5‐mg/ml bovine serum albumin (BSA) solution. The cells were then incubated overnight at 4°C with one of the following rabbit antibodies (diluted 1:100): anti‐β‐catenin, anti‐HIF‐1α or anti‐VEGF (Abcam, Cambridge, UK). The secondary antibody was rhodamine‐conjugated anti‐rabbit antibody (diluted 1:200, Invitrogen, USA), and the nuclei were stained with DAPI. Samples were visualized by CLSM, and images were obtained using a digital camera.

### 4.10 Preparation of the diabetic rat model

We used 36 male Sprague Dawley (SD) rats (12 weeks old; 300–400 g) in this study. Streptozotocin (STZ) was prepared in 0.1 M phosphate citrate buffer (pH 4.5). Freshly prepared STZ (Sigma‐Aldrich) was used to induce diabetes by intraperitoneal injection (60 mg/kg). The rats were examined on the third day after STZ administration to confirm that diabetes had been initiated. Blood was collected from the tail vein of the SD rats and a blood glucose level >250 mg/dl (13.9 mmol/L) measured with a blood glucose meter (Roche) was considered to indicate diabetes. Two weeks after the induction of diabetes, the blood glucose levels and body weight changes of the rats were measured every day. Once the induction of diabetes had been confirmed in the rat model, the experiment was conducted. Before operation, anaesthesia was performed by intraperitoneal injection of a 3% sodium pentobarbital solution (Sigma‐Aldrich) at a dose of 1.0 ml/kg. The defect area was marked accurately with a pen, and the back was cut under aseptic conditions to prepare a standard full‐thickness skin wound (diameter = 1.8 cm).^57^

### 4.11 Measurement of wound closure

After the operation, a digital camera (Nikon, Tokyo, Japan) was used to take pictures of the wound on days 0, 7 and 14. The edge of each wound was traced and ImageJ was used to measure the wound area. The following formula was used to calculate the degree of wound closure: wound reduction rate = [(A0 − At)/A0] × 100%, where A0 is the initial wound area (t = 0) and At is the wound area at each time interval.^58^

### 4.12 H&E and masson staining and IHC

The rats were sacrificed on the 7th and 14th days after the operation, and tissue containing the wound bed and surrounding healthy skin were collected for further study. The samples were fixed in formalin, dehydrated through a graded ethanol series and embedded in paraffin. The sections (5‐μm thick) were stained with H&E and Masson's trichrome and inspected under an optical microscope. Thin‐layer IHC staining of CD34 (1:100, Abcam) was performed to study angiogenesis during wound healing. For IHC staining, the sections were rehydrated, processed to recover the antigen and incubated with the primary antibody at 4°C overnight. Subsequently, a biotinylated secondary antibody and avidin–biotin–peroxidase complex were applied, and the stained sections were visualized using DAB substrate. The sections were then counterstained with haematoxylin, mounted and analysed under an optical microscope.

### 4.13 Statistical analysis

All data are expressed as the mean ± standard deviation (SD). The independent samples *t* test was used to compare the means of two different groups. GraphPad Prism 9 software was used with one‐way analysis of variance (ANOVA) to determine the level of significance, and a *p* value < 0.05 was considered to indicate statistical significance.

## CONFLICT OF INTEREST

The authors declare no competing financial interests.

## AUTHOR CONTRIBUTION

S. Ma and H. Hu are co‐first authors of the article, and they contributed to this work equally. S. Ma and H. Hu designed the experiments. S. Ma and H. Hu performed the experiments and analysed data. H. Hu wrote the manuscript. J. Wu, X. Li, X. Ma, Z. Zhao, Z. Liu, C. Wu, B. Zhao, Y. Wang and W. Jing are responsible for the review and revision of the initial draft.

## Supporting information

Figure S1‐S8

## Data Availability

The [DATA TYPE] data used to support the findings of this study are available from the corresponding author upon request.
