# Correction to “Functional extracellular matrix hydrogel modified with MSC‐derived small extracellular vesicles for chronic wound healing”

**DOI:** 10.1111/cpr.13553

**Published:** 2023-11-08

Ma S, Hu H, Wu J, et al. Functional extracellular matrix hydrogel modified with MSC‐derived small extracellular vesicles for chronic wound healing. *Cell Prolif*. 2022;55:e13196. doi:10.1111/cpr.13196

This note aims to correct two errors of Figure 3B and Figure 8B in the version of this article initially published. The Figure 3B mislabelled the legend name. The immunohistochemical result of Figure 8B was misplaced. The other elements of Figures 3 and 8 remain the same, and the interpretation of the results remains unchanged.

The corrected images for Figures 3 and 8 are below.

We apologise for the errors.

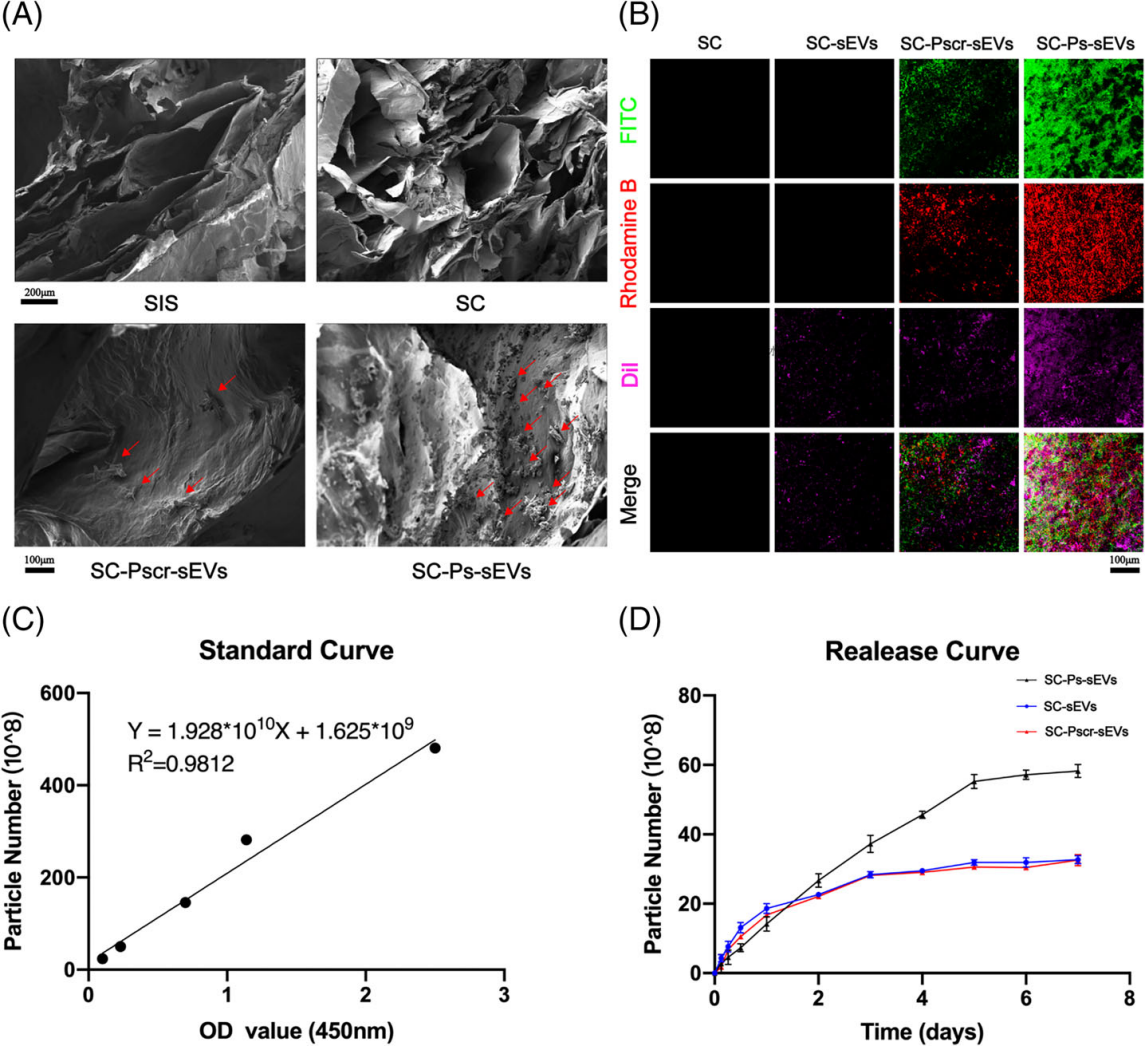

FIGURE 3.

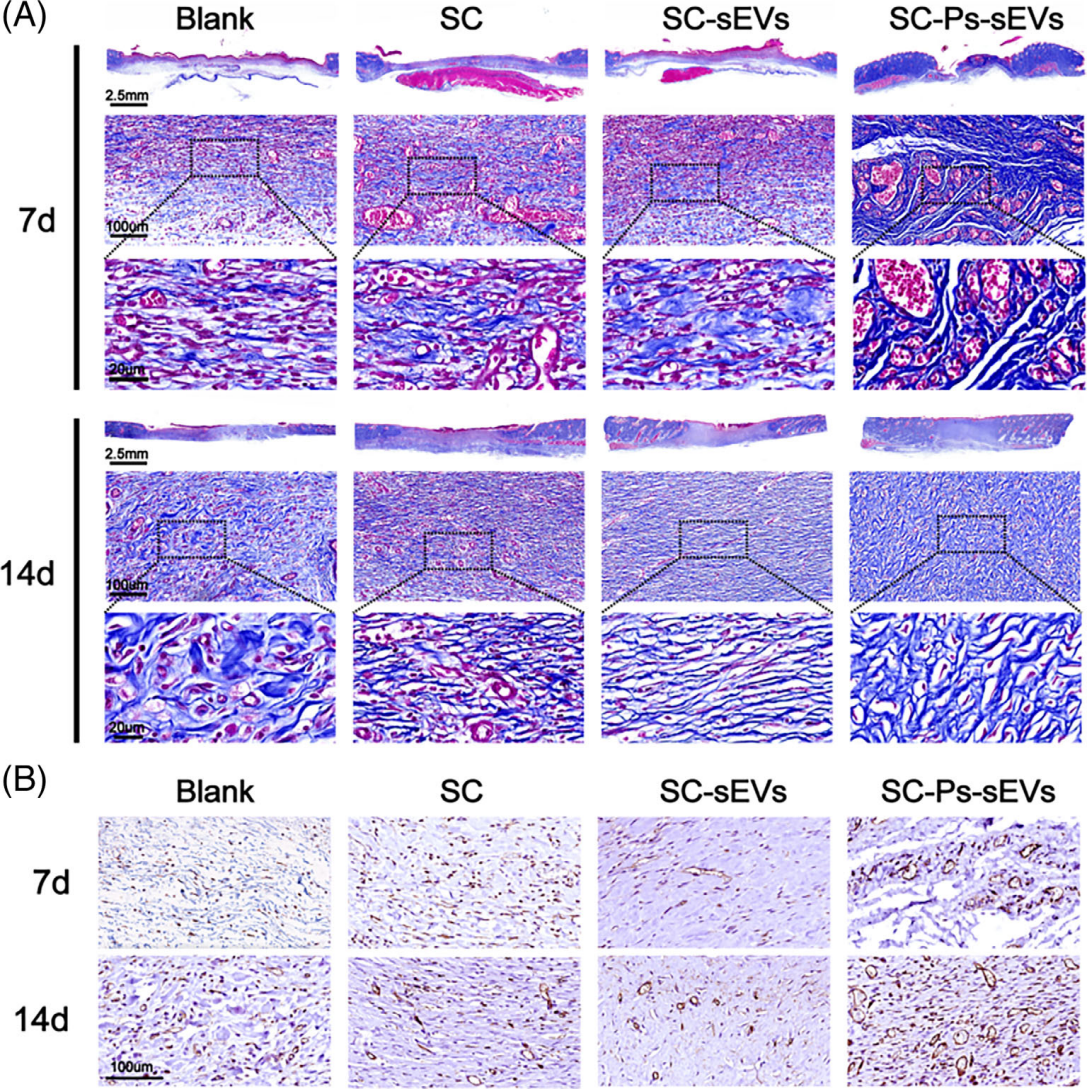

FIGURE 8.

