# Supplementary material for: Functional extracellular matrix hydrogel modified with MSC‐derived small extracellular vesicles for chronic wound healing
Source: Cell Prolif. 2022 Feb 14;55(4):e13196. doi: 10.1111/cpr.13196 (PMC9055911; doi:10.1111/cpr.13196)
Supplement: Supplementary file 1 — Figure S1‐S8 [file CPR-55-e13196-s001.docx]

Supplementary figures for article

Functional Extracellular Matrix Hydrogel Modified with MSCs-Derived Small Extracellular Vesicles for Chronic Wound Healing

Supplementary figures and legends


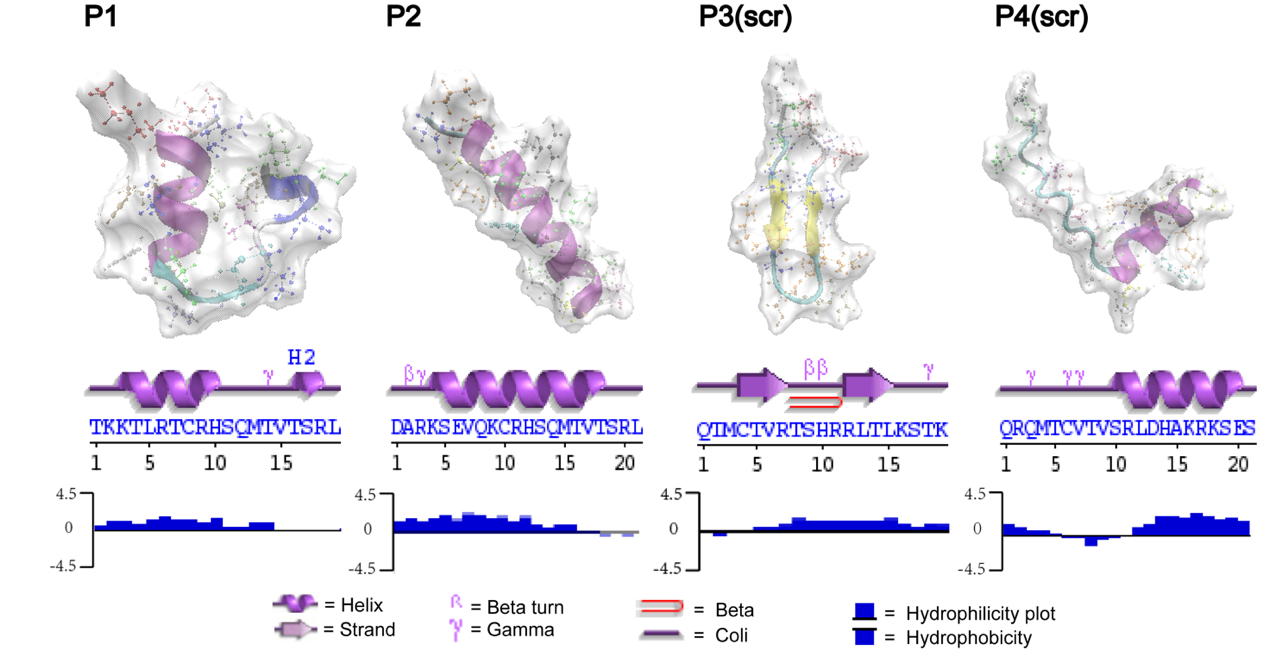


**Figure S1**. Pseudo-three-dimensional views of the molecular architecture. The 3D models were predicted based on their secondary structure (helix, beta turn, beta hairpin, strand, gamma turn and coil). The hydrophilic/hydrophobic distributions shows the properties of the 4 peptides.

P1: TKKTLRTCRHSQMTVTSRL


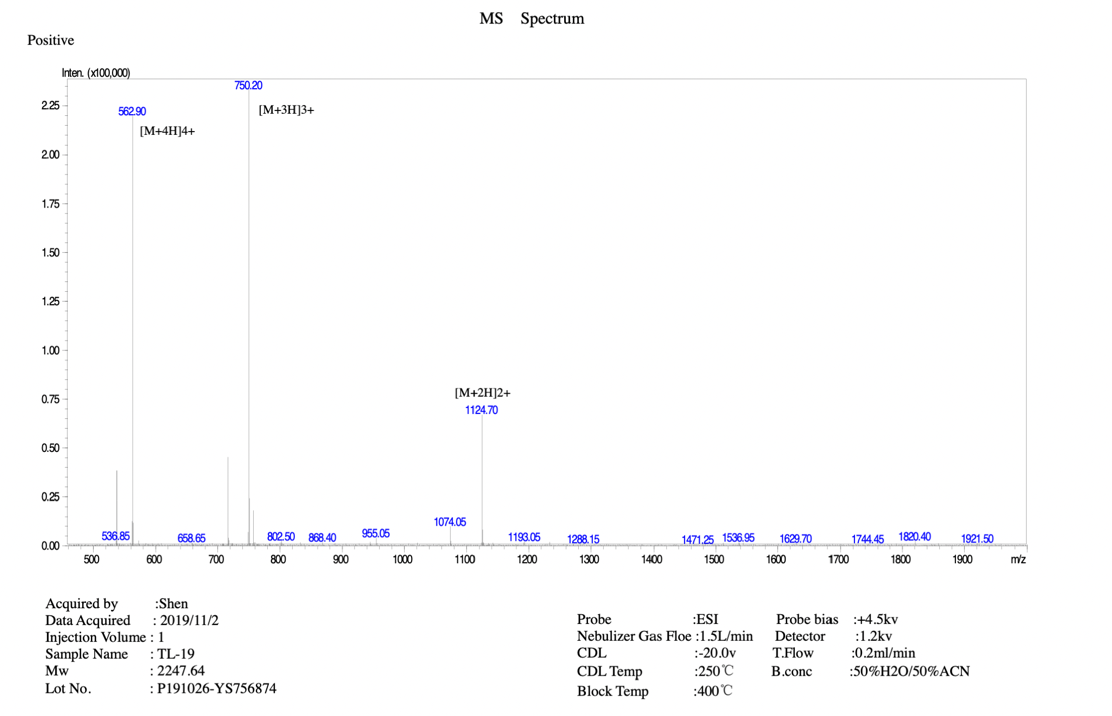


P2: DARKSEVQKCRHSQMTVTSRL


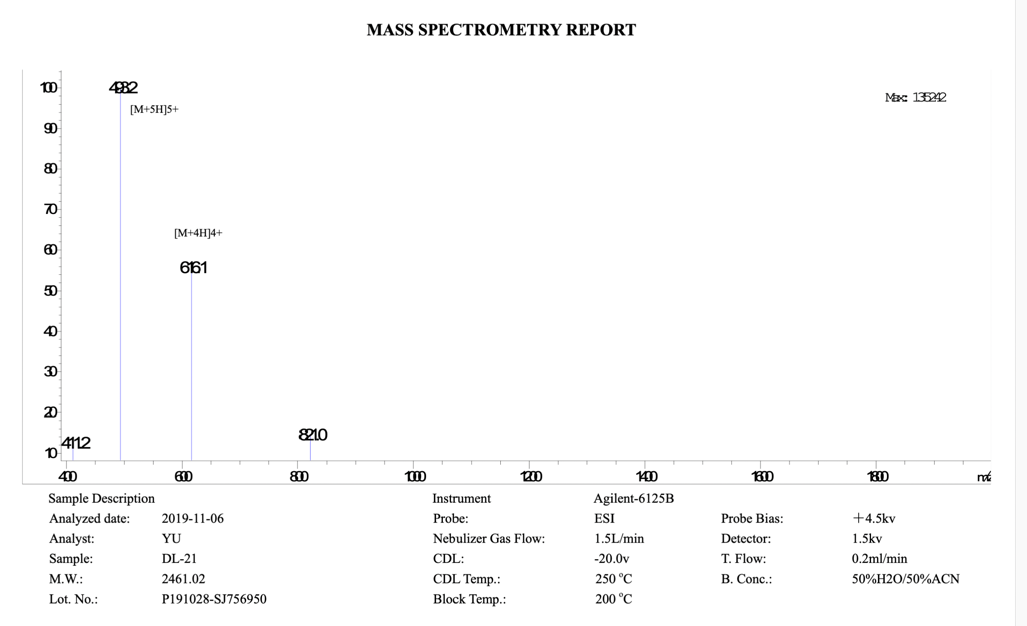


P3(scr): QTMCTVRTSHRRLTLKSTK


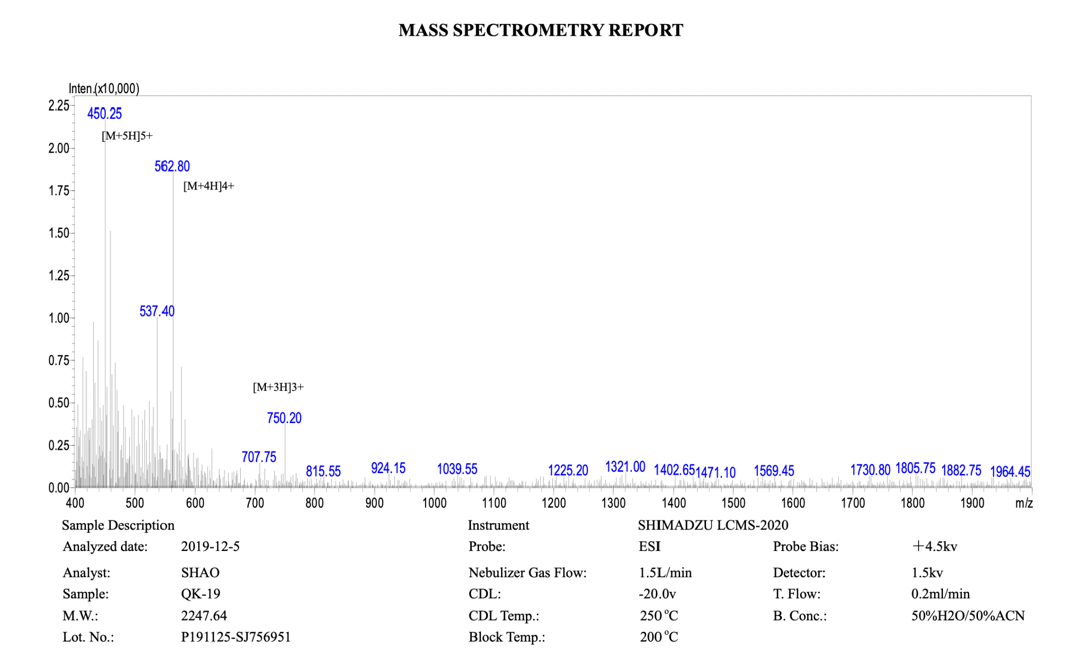


P4(scr): QRQMTCVTVSRLDHAKRKSES


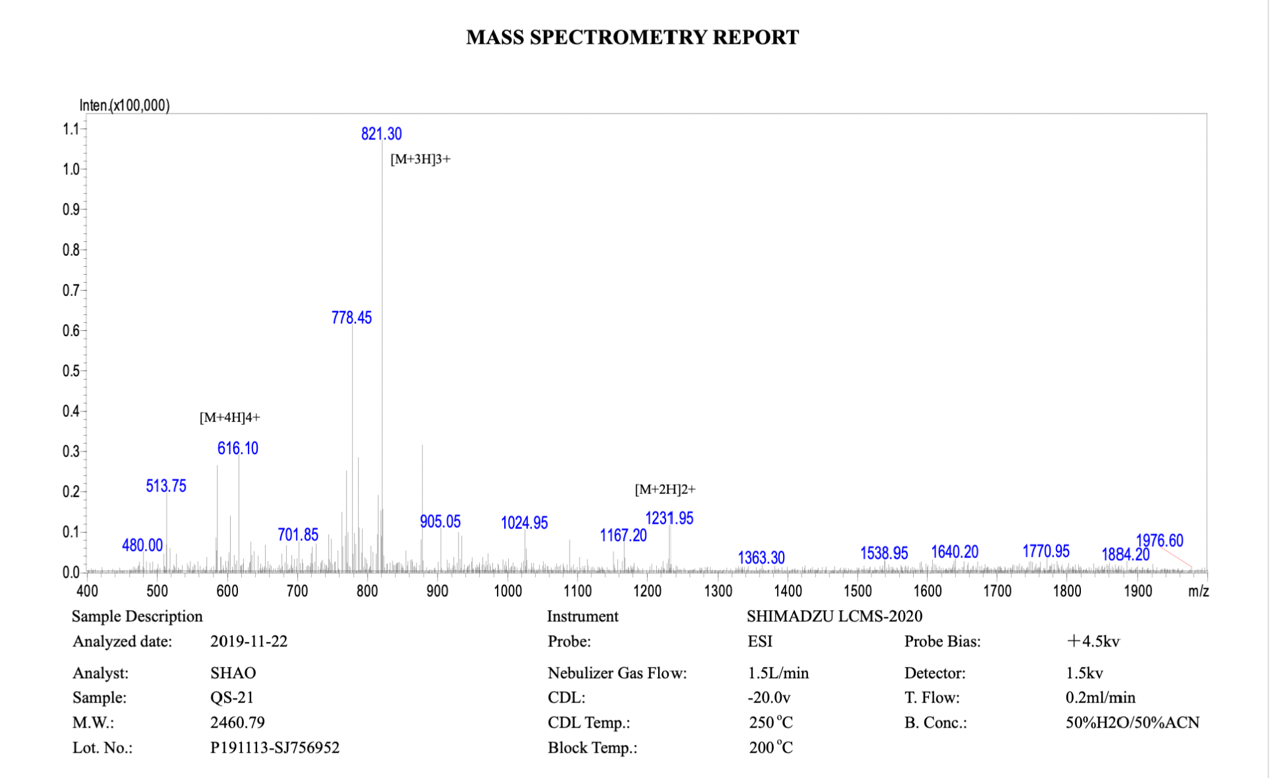


**Figure S2** Mass spectra (MS) of four peptides P1-P4

P1: TKKTLRTCRHSQMTVTSRL

P2: DARKSEVQKCRHSQMTVTSRL


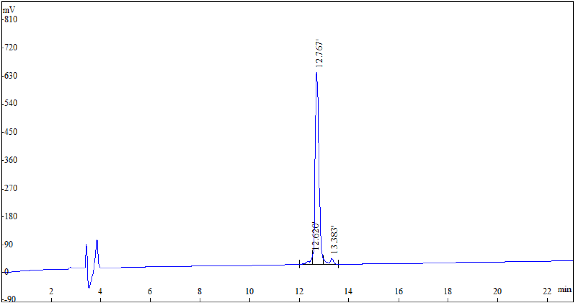


P3(scr): QTMCTVRTSHRRLTLKSTK

P4(scr): QRQMTCVTVSRLDHAKRKSES


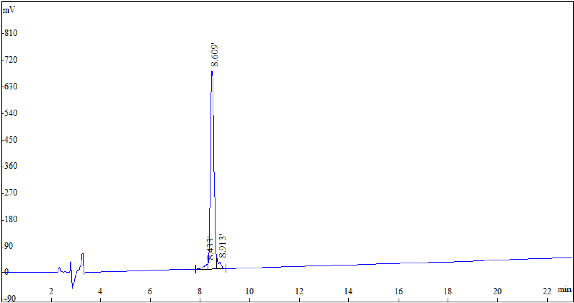


**Figure S3:** High Performance Liquid Chromatography (HPLC) of four peptides: P1-P4


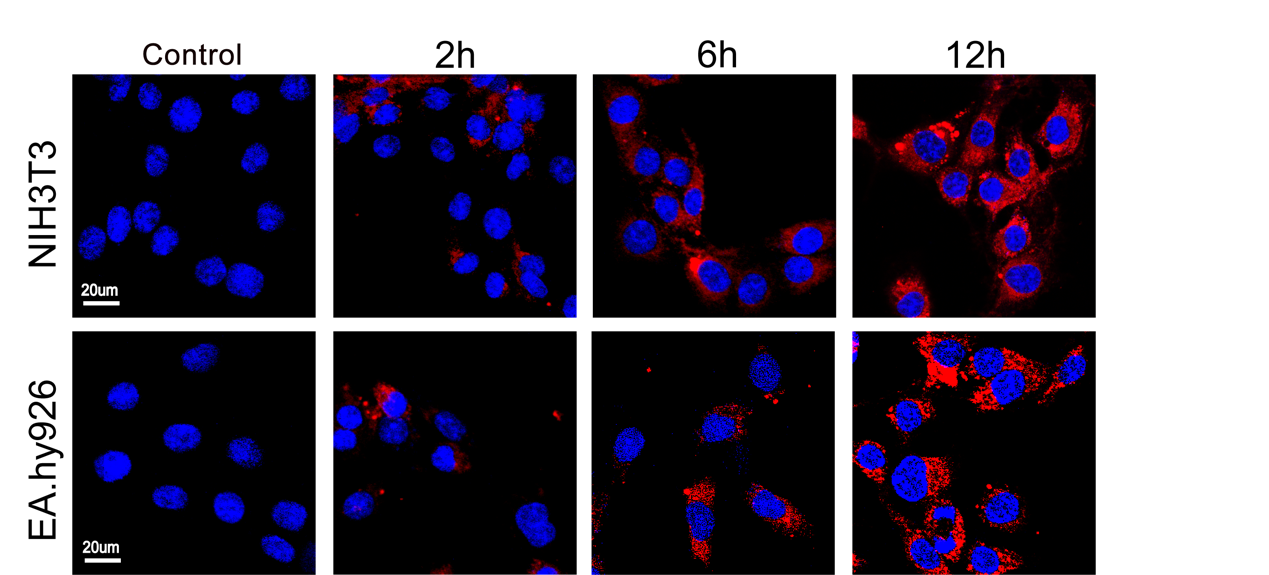


**Figure S4.** Fluorescence microscopy showed the internalization of DiI-labeled ucMSC-derived sEVs by NIH3T3 and EA.hy926 cells after coculture for 2 h, 6 h and 12 h. Nuclei were counterstained with DAPI.


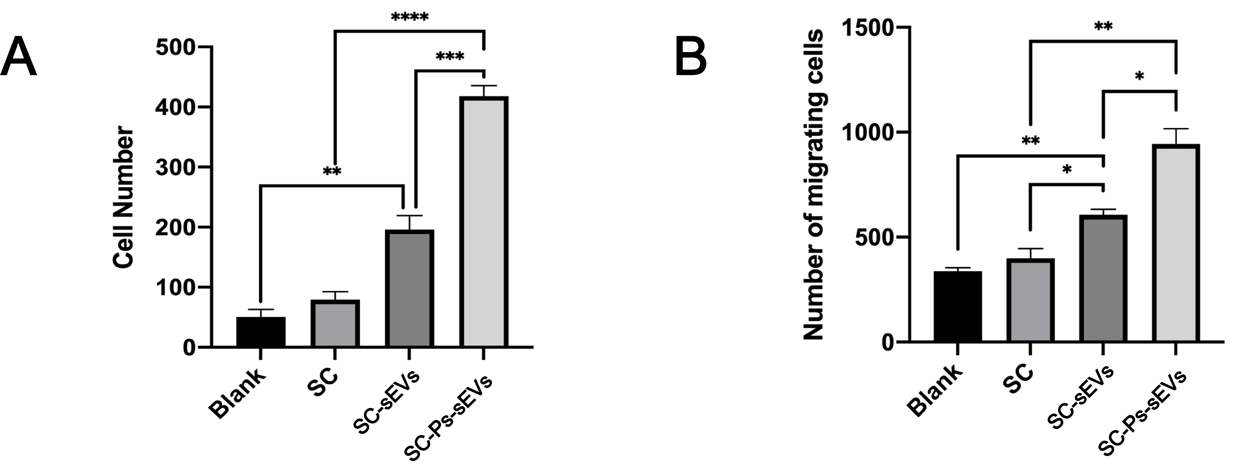


**Figure S5.** (**A**) Quantification of cells in the SYTO 9 staining assay. (**B**) Quantification of NIH3T3 cell migration *p < 0.05; **p < 0.01; ***p <0.005, n=3.


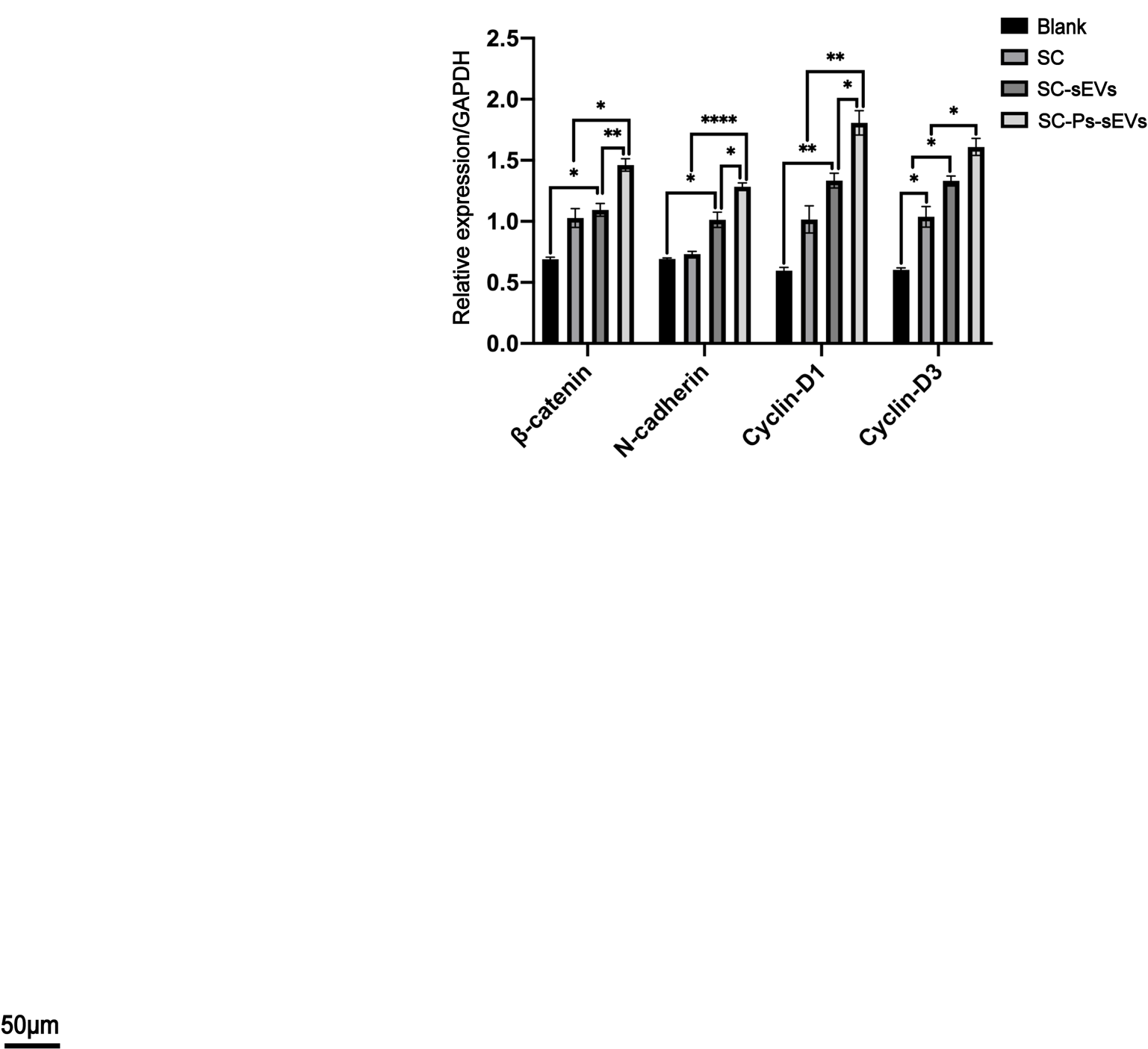


**Figure S6**. Quantitative analysis by western blotting; GAPDH was used as a normalization control. A blank well was used as a negative control, and SC was used as a positive control. *p < 0.05; **p < 0.01; ***p <0.005, n=3.


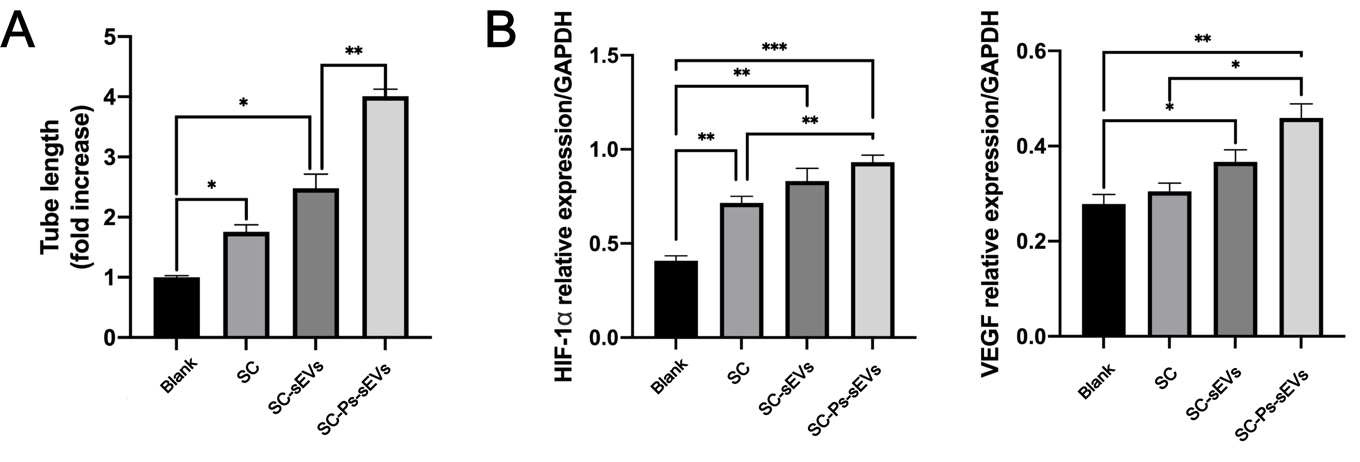


**Figure S7** (**A**) Quantification of tube formation. *p < 0.05; **p < 0.01; ***p <0.005, n=3. (**B**) Quantitative analysis by western blotting; GAPDH was used as a normalization control. A blank well was used as a negative control, and SC was used as a positive control. *p < 0.05; **p < 0.01; ***p <0.005, n=3.


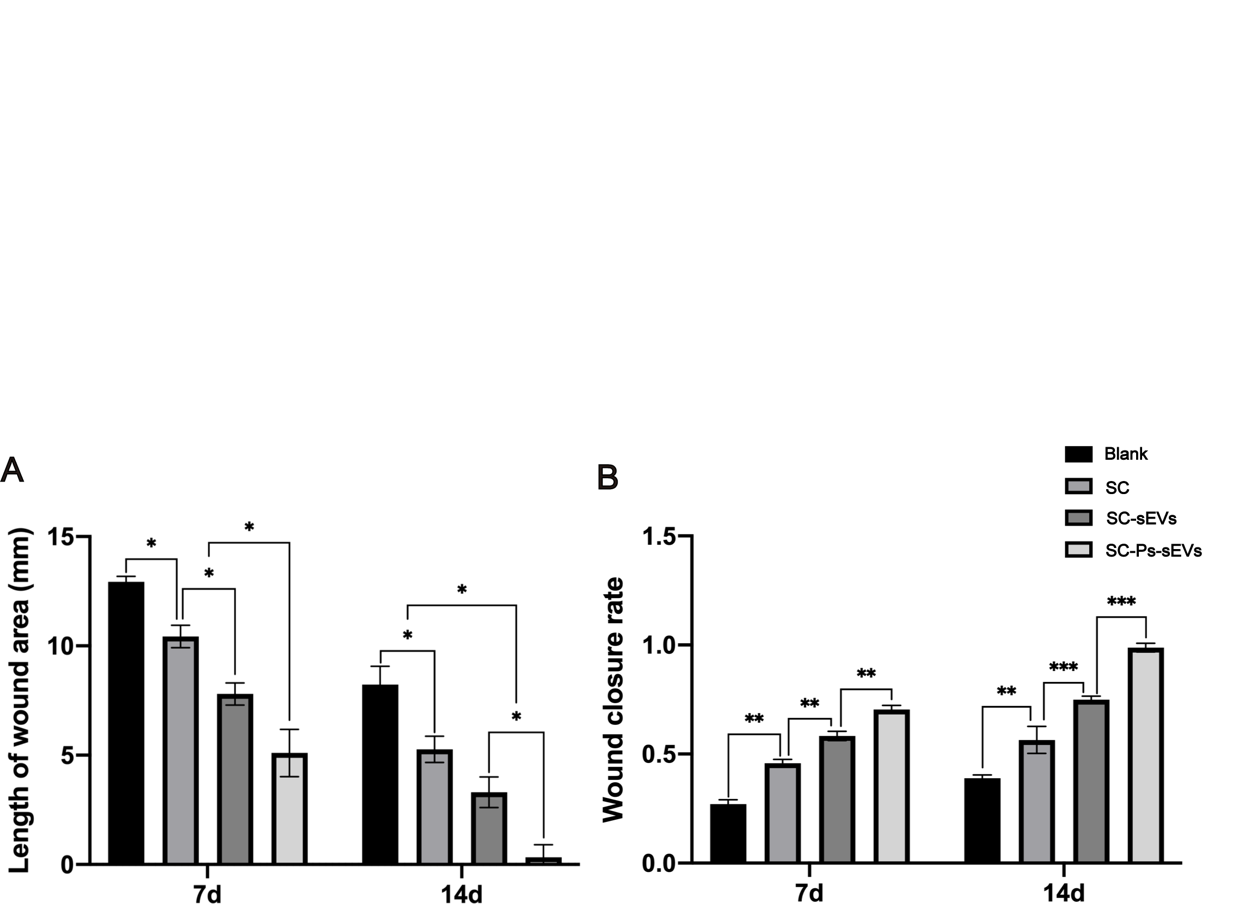


**Figure S8.** (**A**) Quantification of the wound length on days 7 and 14. *p < 0.05; **p < 0.01; ***p <0.005, n=3. (**B**) Wound closure rates at different time points in the four groups. *p < 0.05; **p < 0.01; ***p <0.005, n=3.
